# A 3D Tumor‐Mimicking In Vitro Drug Release Model of Locoregional Chemoembolization Using Deep Learning‐Based Quantitative Analyses

**DOI:** 10.1002/advs.202206195

**Published:** 2023-02-15

**Authors:** Xiaoya Liu, Xueying Wang, Yucheng Luo, Meijuan Wang, Zijian Chen, Xiaoyu Han, Sijia Zhou, Jiahao Wang, Jian Kong, Hanry Yu, Xiaobo Wang, Xiaoying Tang, Qiongyu Guo

**Affiliations:** ^1^ Shenzhen Key Laboratory of Smart Healthcare Engineering Guangdong Provincial Key Laboratory of Advanced Biomaterials Department of Biomedical Engineering Southern University of Science and Technology Shenzhen Guangdong 518055 P. R. China; ^2^ Department of Electronic and Electrical Engineering Southern University of Science and Technology Shenzhen Guangdong 518055 P. R. China; ^3^ Department of Molecular Cellular and Developmental Biology (MCD) Centre de Biologie Integrative (CBI) University of Toulouse CNRS UPS Toulouse 31062 France; ^4^ Mechanobiology Institute National University of Singapore Singapore 117411 Singapore; ^5^ Department of Interventional Radiology First Affiliated Hospital of Southern University of Science and Technology Second Clinical Medical College of Jinan University Shenzhen People's Hospital Shenzhen Guangdong 518020 P. R. China; ^6^ Department of Physiology Institute of Digital Medicine and Mechanobiology Institute National University of Singapore Singapore 117593 Singapore; ^7^ Jiaxing Research Institute Southern University of Science and Technology Jiaxing Zhejiang 314000 P. R. China; ^8^ Department of Pharmacy Shenzhen Children's Hospital Shenzhen Guangdong 518026 P. R. China

**Keywords:** 3D drug release model, decellularized organ, extracellular matrix, hepatocellular carcinoma, transarterial chemoembolization

## Abstract

Primary liver cancer, with the predominant form as hepatocellular carcinoma (HCC), remains a worldwide health problem due to its aggressive and lethal nature. Transarterial chemoembolization, the first‐line treatment option of unresectable HCC that employs drug‐loaded embolic agents to occlude tumor‐feeding arteries and concomitantly delivers chemotherapeutic drugs into the tumor, is still under fierce debate in terms of the treatment parameters. The models that can produce in‐depth knowledge of the overall intratumoral drug release behavior are lacking. This study engineers a 3D tumor‐mimicking drug release model, which successfully overcomes the substantial limitations of conventional in vitro models through utilizing decellularized liver organ as a drug‐testing platform that uniquely incorporates three key features, i.e., complex vasculature systems, drug‐diffusible electronegative extracellular matrix, and controlled drug depletion. This drug release model combining with deep learning‐based computational analyses for the first time permits quantitative evaluation of all important parameters associated with locoregional drug release, including endovascular embolization distribution, intravascular drug retention, and extravascular drug diffusion, and establishes long‐term in vitro–in vivo correlations with in‐human results up to 80 d. This model offers a versatile platform incorporating both tumor‐specific drug diffusion and elimination settings for quantitative evaluation of spatiotemporal drug release kinetics within solid tumors.

## Graphical abstract

A 3D drug release model combined with deep learning‐based computational analyses enables quantitative evaluation of spatiotemporal drug release behaviors within tumor tissues, showing the promise for use as a powerful research tool to facilitate the development and optimization of translational drug compositions while leveraging comprehensive information of the whole drug release process from minimum organ samples for various locoregional treatments.

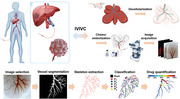

## 1 Introduction

Primary liver cancer, the third lethal cancer that annually causes over 830 000 deaths worldwide, remains a severe health challenge with continuously increasing incidence and mortality.^[^ ^1^, ^2^ ^]^ Hepatocellular carcinoma (HCC), the predominant liver cancer form accounting for 90% of the cases, usually shows hypervascularity especially at medium to late stages.^[^ ^3^, ^4^ ^]^ Transarterial chemoembolization (TACE), which takes advantage of the tumor feeding arterial system by directly injecting drug‐loaded embolic agents into the vasculature to locally derive both embolization and chemotherapy effects for the tumor, has become the mainstay treatment for unresectable HCC over the past two decades.^[^ ^5^, ^6^, ^7^ ^]^ However, conventional TACE (cTACE), which applies liquid‐based water/oil emulsion prepared by physically blending ethiodised oil (EO) with aqueous drug solution, exhibits high rate of adverse effects due to the instability of the emulsion causing large amount of drug instantaneously released into the systemic circulation right after embolization.^[^ ^8^ ^]^ Recently, drug‐eluting beads (DEB)‐based TACE treatment (DEB‐TACE), which employs embolic microspheres electrostatically absorbed with chemotherapy drugs to enhance the drug release controllability, successfully reduces some postembolization syndromes but presents controversial results on the tumor response and patient survival as compared to the cTACE.^[^ ^9^, ^10^ ^]^ Despite wide adoption and numerous clinical trials of both TACE treatments, how the different pharmaceutical embolic agents respond to the HCC intratumoral environment remains elusive and the selection of TACE type as well as treatment parameters is still under fierce debate.^[^ ^11^ ^]^

Unfortunately, there still lacks appropriate models to effectively evaluate drug release kinetics within the tumor tissues for TACE treatment. The in vivo drug distribution information obtained from animal models and clinical samples largely relies on tumor fragments collected at predetermined time intervals, hardly providing in‐depth knowledge of the overall drug release behavior inside the tumor.^[^ ^12^, ^13^, ^14^ ^]^ Current in vitro drug release characterization methods based on dialysis bags,^[^ ^15^ ^]^ flow‐through cell,^[^ ^16^ ^]^ and vascular flow system^[^ ^17^ ^]^ are mainly designed to estimate systemic drug release kinetics, but unable to provide information of the pharmacokinetics inside the tumor tissues. Very recently, Caine et al. developed a tissue‐mimic phantom consisting of a single alginate‐agarose hydrogel‐based channel, which was fully packed with drug‐eluting beads for drug diffusion assessment under a static condition.^[^ ^18^ ^]^ Nevertheless, this model utilized an over‐simplified platform and failed to incorporate crucial drug elimination factors caused by potential cellular metabolism and/or drainage into neighboring vessels inside the tumor, producing drug release results barely comparable to the in vivo responses.

Therefore, developing physiologically relevant in vitro models that can incorporate both tumor‐specific drug diffusion and elimination settings to evaluate locoregional performance of the TACE embolic agents is urgently needed for the advancement, refinement, and standardization of the embolus formulations.^[^ ^10^, ^19^ ^]^ Decellularized organ scaffolds, which can preserve both composition and architecture of native tissues’ extracellular matrix (ECM) upon decellularization processes, present an ideal platform for this task.^[^ ^20^, ^21^ ^]^ Such acellular scaffolds have been widely applied in tissue engineering and regeneration applications with specific focus on cell–ECM interactions, but is seldomly explored as drug release templates for pharmaceutical characterizations.^[^ ^22^, ^23^ ^]^ Here we propose a decellularized liver organ‐based 3D in vitro drug release model to quantitatively analyze the spatiotemporal drug delivery pattern of the TACE treatment. This model uniquely resembles the key features of in vivo drug release conditions: 1) maintaining complex vasculature system for loading drug‐eluting embolic agents, 2) preserving hepatic‐specific ECM for extravascular drug diffusion, and 3) keeping continuous perfusion flow throughout the model to control drug depletion. To demonstrate the feasibility of our method, we quantitatively evaluate and compare the two commonly used chemoembolization agents used in cTACE and DEB‐TACE, respectively, as well as a drug solution control. We specifically monitor the drug release behavior up to 80 d and for the first time establish a long‐term in vitro–in vivo correlations (IVIVCs) between in vitro drug responses and clinical results.

Quantification of intratumoral drug release patterns involving intravascular drug retention and extravascular drug diffusion of hundreds of drug‐containing vascular branches can be time‐consuming and labor‐intensive if conducted manually. Despite of recent advances in deep learning‐based automatic vessel segmentation, there has been no attempt on computational analyses of drug release‐associated vessel features, not to mention more sophisticated drug‐related topological characteristics.^[^ ^24^, ^25^, ^26^ ^]^ Here we for the first time develop a customized and semiautomated pipeline to quantitatively examine the progression of the spatial drug diffusion pattern over time. We design a deep learning‐based pipeline mainly comprising a dual‐attention U‐Net trained generative adversarial network (DAU‐GAN), which incorporates GAN and attention mechanisms to capture the vessel distributions through strengthening both spatial and channel features, for the vascular segmentation.^[^ ^27^ ^]^ With the accurately segmented vessel mask, we further extract the vessel skeletons to analyze the topological characteristics of drug‐contained vessels, including length, diameter, and branching degree. The intravascular drug retention mapping of the local chemotherapy is obtained based on the quantitative relationship between the drug concentration, vessel diameter, and the grayscale of the image. Utilization of computational analyses in the 3D in vitro drug release model not only allows for an in‐depth understanding of locoregional drug responses for the treatment modality, but also enables facile comparison of various locoregional therapeutics for preclinical pharmaceutical characterizations.

## 2 Results

### 2.1 Decellularized Liver as Organ‐Structured Drug Release Model for Local Chemotherapy Evaluation

**Figure 1:**
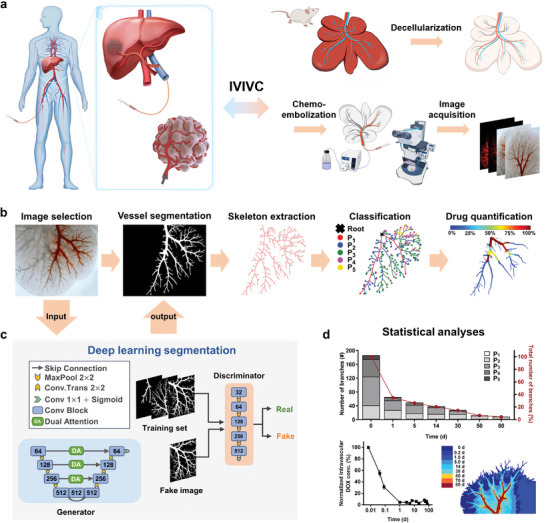
Workflow of organ‐structured drug release model development and deep learning‐assisted computational analyses. a) Construction of decellularized rat liver‐based drug release model to establish IVIVC with in‐human results in TACE. In the 3D drug release model, a drug‐loaded embolic agent is injected into one main branch of portal vein vasculature to mimic superselective treatment of TACE while a buffer solution is constantly perfused through the hepatic vein to control drug depletion. b) Outline of computational pipeline for spatiotemporal drug release quantification that consists of four steps: (i) inputting original images into the proposed DAU‐GAN and generating binary segmentation results, (ii) extracting vessel skeleton from the segmentation results, (iii) classifying the vessel branches into five levels, and (iv) deriving intravascular drug concentration maps of Levels P_1_–P_3_ according to the diameter and grayscale of the vessel branches. c) Schematic of the DAU‐GAN pipeline employing the general framework of GAN that consists of a generator, which applies a U‐Net structure complemented with a dual attention (DA) module on each Skip Connection path, and a discriminator. The number in Conv Block denotes the number of the corresponding feature map's channels. d) Subsequent statistical analyses based on the imaging results obtained in (b).

A 3D tumor‐mimicking drug release model based on decellularized liver matrix (DLM) was developed to establish an effective IVIVC for TACE treatment (**Figure** **1**a). The acellular liver was obtained by perfusing fresh rat liver with detergent solutions of 4% Triton X‐100/0.02% EGTA solution and 0.5% sodium dodecyl sulfate (SDS) aqueous solution following a modified decellularization protocol (Figure S1, Supporting Information).^[^ ^28^ ^]^ The acellular liver was then fixed with 4% paraformaldehyde overnight to achieve optimized long‐term stability of physical shape and optical transparency (20 ± 3% in transmittance). This DLM model contains hepatic‐specific intact vasculatures and extracellular matrix, providing a unique template for drug release test. A variety of clinically relevant doxorubicin‐loaded embolic agents can be directly injected into the portal vein of the DLM model due to the comparable vasculature dimensions with those in distal arteries of human HCC.^[^ ^28^, ^29^, ^30^ ^]^ The spatiotemporal drug release kinetics were evaluated with continuous flow of aqueous solution through the hepatic vein to mimic the tumor‐specific drug depletion condition. Doxorubicin, one of the most frequently applied anticancer drugs in TACE, was tested.^[^ ^3^, ^31^, ^32^ ^]^ The red color and strong fluorescence of doxorubicin combined with the excellent optical transparency of the DLM model enables a direct observation of the drug distribution using bright‐field and fluorescent microscopies.

Quantitative evaluations of the spatiotemporal drug diffusion dynamics in the DLM model were performed through deep learning‐based vascular segmentation, skeleton extraction and classification, as well as drug statistical analyses (Figure 1b). Accurate segmentation of drug‐containing vessels is particularly important but challenging due to the weak contrast between drug‐containing and nondrug‐containing vessel pixels, fuzzy vessel boundary caused by drug diffusion, and limited sample size of the training dataset. To tackle this problem, we designed DAU‐GAN that employs the following specific strategies to enhance the segmentation performance (Figure 1c). In the deep learning pipeline, we applied the general framework of GAN containing a generator (G) and a discriminator (D) that were trained alternatively to make the predicted output indistinguishable from the ground truth. The G adopts an encoding‐decoding U‐Net structure, which is especially complemented with a DA module comprising a channel attention block and a spatial attention block on each Skip Connection path to endow excellent generation capability. Importantly, GAN‐based segmentation can improve model generalization with small amount of image samples, and the dual attentions emphasize the spatial and channel features to have a better feature representation for solving the low contrast of vessel images. Considering that our subsequent skeleton extraction precision relies heavily on the segmentation accuracy and even deviation of a few pixels may lead to wrong predictions of topological quantification, the automatically predicted segmentation results were postprocessed with some corrections when necessary. Our semiautomatic annotation scheme took an average of 5 min to segment a single image, saving several hours per image over fully manual annotation.

We then deployed a thinning algorithm for each segmentation result to delete redundant pixels until there left with a single‐pixel width skeleton line (Figure 1b). For the single‐pixel skeleton, we designed a vessel classification rule which automatically divided the vascular skeleton into five levels (P_1_ to P_5_) from main to distal for further topological analyses (Figure S2, Supporting Information). Specifically, the P_1_‐level vessels were defined as the main vessels extending from the vascular root, and the P_N_‐level vessels were defined as the vessel branches stemming from the P_N‐1_‐level vessels. In this way, we iteratively identified all five‐level vessels. Since the embolic agents barely reach vessel branches above Level P_5_, all vessels above Level P_5_ were considered as Level P_5_ as well. After vessel classification, we then fitted a drug concentration model according to the grayscale of the image and the vascular diameter. This allows us to visualize and quantify changes in drug concentration inside the vessels over time. Through the entire pipeline, we ended up with various characteristics of the drug diffusion process, including the numbers, lengths, and drug concentration degrees of different levels’ vessels at different time points (Figure 1d). Clearly, the deep learning‐based vessel segmentation module laid the foundation for the quantification of the spatiotemporal drug release behavior in DLM.

### 2.2 Performance of Deep Learning‐Based Vessel Segmentation

**Figure 2:**
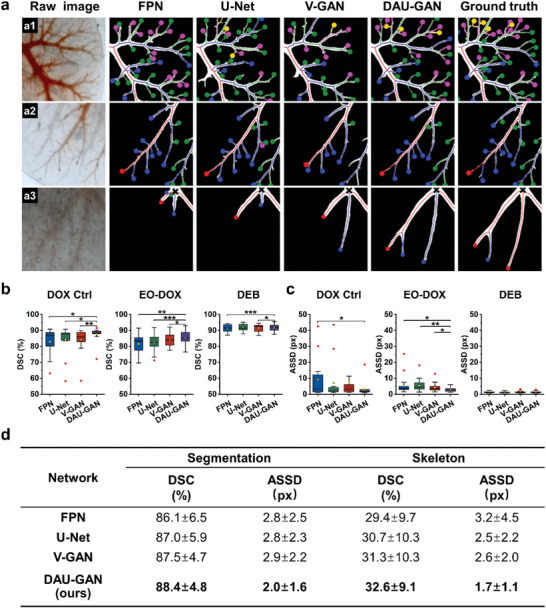
Evaluations of deep learning‐based vessel segmentation and skeleton extraction in DLM. a) Representative segmentation and topology results obtained from different methods. The binary images represent the segmentation results, the colored lines illustrate the skeletons, and the colored marks (consistent with the classification given in Figure 1b) indicate the topology classification results. b,c) Box plots of DSC (b) and ASSD (c) on DOX Ctrl, EO‐DOX, and DEB's segmentation results. The central lines demonstrate the median values, the yellow points show the average values, the boxes represent the interquartile ranges, the whiskers exhibit the smallest and largest values, and the red points mark the outliers. Two‐sided paired *t*‐test was performed to compare groups (*n* = 20 replicates). *P < 0.05, **P < 0.01, and ***P < 0.001. d) Quantitative comparison results of DSC and ASSD for segmentation and skeleton analyses. Bold represents the best performance.

We first performed deep learning‐based segmentation of drug‐containing vessels in DLM for the TACE evaluation (**Figure** **2**a). Three drug formulations, including EO‐based doxorubicin emulsion (EO‐DOX), doxorubicin‐loaded DEB as well as a doxorubicin solution control (DOX Ctrl), were selectively injected into portal venous vasculature of DLM and long‐term drug release of these formulations were monitored and compared. The recorded images were then fed into DAU‐GAN to automatically predict the segmentation results.

To demonstrate the superiority of our proposed DAU‐GAN, we compared its performance with those of another three neural networks, namely, feature pyramid network (FPN),^[^ ^33^ ^]^ U‐Net,^[^ ^34^ ^]^ and V‐GAN.^[^ ^35^ ^]^ The two deep learning pipelines such as FPN and U‐Net have been widely applied in medical image segmentation, and V‐GAN further combines U‐Net with GAN to improve the segmentation through an adversarial training process. As shown in Figure 2a, all these four networks performed well in segmenting high‐intensity regions, clearly suggesting the power of deep learning‐based segmentation. As for relatively low‐intensity images, DAU‐GAN exhibited higher accuracy than the other three methods, which led to DAU‐GAN's topology being closer to the ground truth. Note that differences of even a few pixels in the skeleton could lead to substantial changes in the topology (the a3 row of Figure 2a), and therefore the topology analysis pipeline relied heavily on very accurate segmentation results. As such, we corrected the automatically predicted segmentation results when necessary, making our entire segmentation pipeline semiautomatic. This process nevertheless is still more computationally efficient and user friendly than the fully manual manner.

Quantitative evaluations of the four different methods, in terms of the dice similarity coefficient (DSC) and the average symmetric surface distance (ASSD), were further conducted for both the segmentation and the skeleton results obtained from all images acquired under the three drug formulations (Figure 2b–d). DSC describes pixel‐level similarity between the automatically predicted result and the ground truth, and ASSD quantifies surface boundary distance between the automatically predicted result and the ground truth. We conducted statistical comparisons on the segmentation and skeleton extraction performances on each of the three sets of images, namely, DOX Ctrl, EO‐DOX, and DEB (Figure 2b,c; Figure S3, Supporting Information). DAU‐GAN significantly outperformed all other three compared methods especially for DOX Ctrl and EO‐DOX. For DEB, all methods performed very well with relatively high segmentation accuracies since the drug‐diffusion pattern was very stable in the vessels along time. Apparently, despite of the different segmentation difficulties of images acquired under the three anticancer formulations, DAU‐GAN demonstrated a very strong generalization ability and was significantly better than the other three methods.

### 2.3 Quantification of Intravascular Drug Distribution

**Figure 3:**
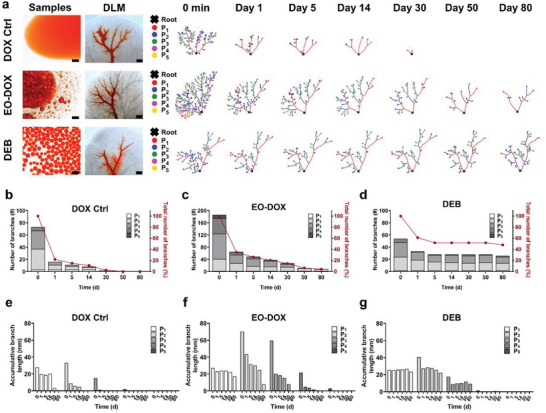
Vessel topological analyses. a) Skeleton extraction and classification of drug‐containing vessels of three drug formulations, i.e., DOX Ctrl, EO‐DOX, and DEB, tested in the DLM model over 80 d. Morphologies of the embolic agents (Scale bar: 200 µm) and the initial appearances of the corresponding DLM model (Scale bar: 2 mm) are also exhibited. b–g) Quantification of the number of branches (b–d) and accumulative branch length (e–g) of the three drug formulations during the drug release test period. P_1_–P_5_ represent five levels of vessel branches according to our classification methods.

Based on deep learning‐assisted vascular segmentation and topology analyses, we evaluated the progression of intravascular drug distribution of the three drug formulations, EO‐DOX, DEB, and DOX Ctrl, up to 80 d (**Figure** **3**a; Figure S4 and Tables S1–S6, Supporting Information). Both EO‐DOX and DEB groups employed the same formulations widely applied in TACE treatments,^[^ ^10^, ^36^ ^]^ while DOX Ctrl was used as a control group. Note that DOX Ctrl is also employed in another type of locoregional chemotherapy, i.e., hepatic arterial infusion chemotherapy, which directly delivers chemotherapeutic drug solution into tumor to treat advanced‐stage HCC.^[^ ^37^, ^38^ ^]^ The EO‐DOX, which was prepared by mixing EO with aqueous doxorubicin solution (5.0 mg mL^−1^) at a volume ratio of 2:1, clearly exhibited desired water‐in‐oil emulsion type. The DEB electrostatically loaded with 25 mg mL^−1^ doxorubicin demonstrated spherical shape with an average diameter of 98 ± 3 µm. To compare, the DOX Ctrl with the same drug concentration as the aqueous phase of EO‐DOX (i.e., 5.0 mg mL^−1^) was also tested. Note that instead of testing the same drug concentrations in the embolic agents of EO‐DOX and DEB, we selected the clinically relevant drug formulations frequently employed in human patients to provide in‐depth understanding of the intratumoral drug release behaviors in clinical settings.

Each of the three drug formulations was selectively injected through one main branch of the portal vein to mimic the local chemotherapy in TACE procedures.^[^ ^39^, ^40^ ^]^ Due to the optical transparency of DLM, the intravascular penetration depth and embolization endpoint of embolic agents are directly visualizable. The injection of the DOX Ctrl group was carefully managed at a slow speed to maintain the majority of the drug solution inside the vessel branches of Levels P_1_–P_3_ (Figure 3a), while further injection of the drug solution to reach the distal vessels of Levels P_4_–P_5_ could easily lead to severe extravascular perfusion (Figure S5, Supporting Information). Unlike DOX Ctrl, the oil‐based emulsion of EO‐DOX was hydrophobic and well stayed inside the vasculature. The embolization end‐points of EO‐DOX were primarily determined by the materials properties of the emulsion such as emulsion viscosity and stability as well as the injection pressure. Previously, we found that the injection pressure of EO dramatically increased when arriving at the capillary endings of Level P_5_ (dia. 55 ± 7 µm), which could cause embolic side‐effects such as vascular damage and emulsion leaking.^[^ ^28^ ^]^ Accordingly, the EO‐DOX was smoothly injected into the vasculature until reaching vessel branches of Level P_4_ (dia. 64 ± 8 µm) to mimic the clinical conditions. By contrast, the embolization end‐points of DEB were simply dependent on the sizes of the embolic particles (dia. 98 ± 3 µm), leading to the embolic distribution down to Level P_3_’s vessel branches (dia. 100 ± 15 µm) (Figure S6a,b, Supporting Information). In the embolization end‐points, some embolic particles appeared to be disconnected from the main drug‐containing vessels, but clearly exhibited the fluorescent drug diffused from the embolic particles in the gap (Figure S6c, Supporting Information). Thus, the disconnected embolic particles were also included in the drug‐containing vessels.

Right after the injection, constant perfusion of buffer solution, which is applied to control the drug depletion speed, was employed at a rate of 1.0 mL min^−1^ through the hepatic vein. Continuous perfusion of the buffer solution through the hepatic vein was performed to produce tumor‐specific drug depletion condition, which mimics the crucial drug elimination factors caused by potential cellular metabolism and/or drainage into neighboring vessels inside the tumor. In clinical trials, the DEB used in TACE was reported to release doxorubicin extremely slowly with significant amount of drug remaining in the patient's liver tumor even after 80 d.^[^ ^41^, ^42^ ^]^ To gain a good IVIVC, we used pure water, instead of physiological saline solution, as the elution medium for perfusion to extend the release rate of drug‐loaded embolic agents in the DLM model.

The three drug formulations exhibited distinctive drug release behaviors (Figure 3a). For the DOX Ctrl, the intravascular doxorubicin solution was quickly diffused into surrounding tissues within 24 h. After 24 h, less than 20% of vascular branches was detected with doxorubicin (Figure 3b), showing the relative accumulative branch length of Levels P_1_ to P_3_ decreasing from 70.9%, 26.6%, down to 7.8%, respectively, as compared to those at the initial time point (Figure 3e). This indicates that the vascular diameter plays a dominate role in the locoregional drug release rate in terms of the simplified aqueous solution‐based drug formulation. The remaining drug was completely depleted in the main vascular branches within 30 d at a much slower speed.

For the liquid‐based embolic agent of EO‐DOX, we found that the drug was also quickly diffused into surrounding extravascular matrix, showing about 60% of drug‐containing vascular branches disappeared within 24 h (Figure 3c). After that, the remaining drug in the main vascular branches was depleted at a much slower rate than DOX Ctrl, showing significant amount of drug in vessel branches of Levels P_1_–P_2_ detectable even for more than 80 d (Figure 3a,f). Such a drug release behavior could be attributed to instability of the oil–water emulsion, which exhibited phase separation occurring in a few minutes right after the preparation of the emulsion.^[^ ^43^ ^]^ In distal vessels, we directly observed demulsification of EO‐DOX showing oil droplets dissolved with small amount of tiny aqueous drug droplets (Figure S7a, Supporting Information). Therefore, the initial drug burst of EO‐DOX could be mainly attributed to the phase‐separated aqueous phase, whereas the second extended drug release stage was associated with the oil phase, which was detected to adsorb ≈5.0% of the doxorubicin solution when saturated in equilibrium (Figure S7b, Supporting Information).

In contrast to EO‐DOX, the DEB exhibited stable drug‐contained vascular topology throughout the testing time window, indicating that the doxorubicin electrostatically adsorbed in the beads maintained much more sustained release kinetics for more than 80 d (Figure 3a). The embolic beads mainly located in vascular branches of Levels P_1_–P_3_ and only some of them scatteringly reach the distal vessels of Level P_4_. As expected, the unloaded and loosely absorbed doxorubicin was first rapidly released within 24 h, leading to an initial rapid decrease of 40% of the drug‐containing vessel branch quantity in the DEB group (Figure 3d). The embolic beads, which adsorbed the majority of the drug, were extremely stable inside the vessels (Figure 3g). After constantly perfusion over 80 d, the DEB still maintained structural integrity and presented bright color due to high drug content. Therefore, the drug‐eluting microspheres stably occluded the targeted vessels with superior extended drug release in the DLM model, which is in consistent with previous in vitro and in vivo trials.^[^ ^44^, ^45^, ^46^ ^]^ Furthermore, we noticed that the initial drug burst of the first day may be affected by many factors such as injection speed and pressure, yet the latter release stage after one day demonstrated great reproducibility for all three drug formations as confirmed in duplicated studies (Figures S8 and S9, Supporting Information).

### 2.4 Quantification of Extravascular Drug Diffusion and IVIVC

**Figure 4:**
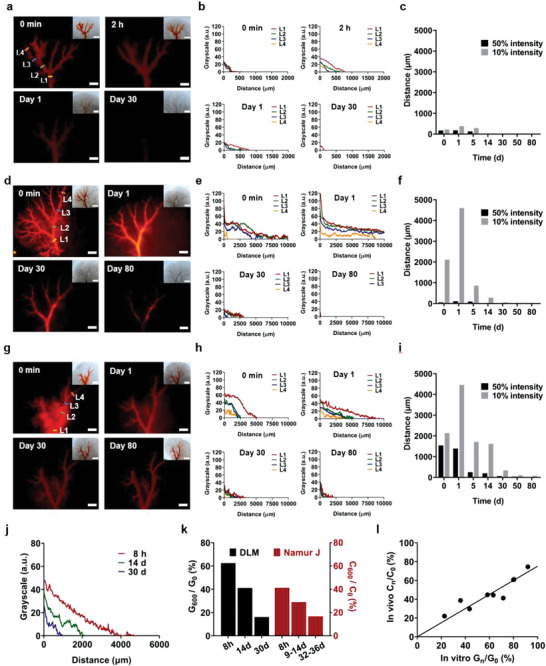
Extravascular drug diffusion in DLM model and IVIVC. Local drug release behaviors of three drug formulations, i.e., a–c) DOX Ctrl, d–f) EO‐DOX, and g–i) DEB. Based on the fluorescent images monitored over 80 d (a,d,g), four vessel locations (L1: 800–1000 µm; L2: 600–800 µm; L3: 400–600 µm; and L4: 200–400 µm) are selected to compare the extravascular drug diffusion depth in different time points (b,e,h). For L2 location, the extravascular drug diffusion depths, which are characterized by the distances corresponding to the grayscale of 10% or 50% as compared to that adjacent to the vessel wall, are compared (c,f,i). The extravascular drug diffusion of j) DEB (L2 location) exhibits comparable release kinetics to k) the in‐human HCC results reported by Namur et al.^[^ ^50^ ^]^, enabling the establishment of an effective IVIVC (*n* = 100, 200, and 600 µm; slope: 0.752; *R* ^2^: 0.983; l).

Taking advantage of the strong self‐fluorescence of doxorubicin at low concentrations, the extravascular drug diffusion depth was determined based on the fluorescent microscopy images (**Figure** **4**). The lower limit of quantitation of doxorubicin solution detectable in the fluorescent images at predetermined microscopy settings was determined to be ≈0.5 µg mL^−1^, which is within the range of the half‐maximal drug inhibitory concentration (IC50) for doxorubicin treatment of HepG2 cells reported previously.^[^ ^15^, ^47^, ^48^, ^49^ ^]^ This indicates that the fluorescently detected area can be a good indicator of effective drug treatment region.

Four vascular locations with varied diameters ranged from 200 to 1000 µm were selected to compare the drug diffusion capability from vessels into surrounding extravascular matrix. Here we define *D*(*n*%) as the extravascular diffusion depth corresponding to the grayscale of *n*% as compared to that adjacent to the vessel wall. In the DOX Ctrl, most doxorubicin was quickly diffused and drained within 24 h (Figure 4a,b). After that, very few doxorubicin was observed outside the vessel, showing *D*(10%) of only 385 µm on Day 1 (Figure 4c). The extravascular fluorescent drug was almost undetectable after 5 d.

Different from the DOX Ctrl, the doxorubicin in EO‐DOX showed greatly enhanced extravascular diffusion capability up to 30 d. The *D*(10%) witnessed a remarkable increase from 2115 µm on Day 0 to 4607 µm on Day 1, which might ascribe to the gradual demulsification of EO‐DOX producing concentrated aqueous doxorubicin solution phase inside the vessels (Figure 4d,e). The intravascular fluorescence intensity of Day 1 was especially strong, which is probably caused by the Tyndall effect in the oil phase dissolved with very fine doxorubicin aqueous droplets (Figure S7a, Supporting Information). After 1 d, the *D*(10%) was then quickly decreased, showing significant extravascular drug diffusion with 14 d (Figure 4f), which is consistent with the extended intratumoral drug delivery of EO‐based embolic emulsions reported in animal results.^[^ ^49^ ^]^ The intravascular doxorubicin could be detected up to 80 d, whereas almost no extravascular diffusion from the EO‐DOX was observed as early as 30 d. In addition, the *D*(50%) was particularly low, showing less than 110 µm throughout the drug release test, indicating that the drug concentration rapidly dropped with the extravascular diffusion depth (Figure 4f).

Compared to EO‐DOX, the DEB further improved the extravascular drug diffusion capability with continuously release from the beads over the entire drug release test (Figure 4g,h). An obvious drug release from DEB due to unloaded doxorubicin was captured instantly right after injected into DLM, which is consistent with previous work.^[^ ^17^ ^]^ We could easily detect the vessel wall of drug containing branches despite of the high fluorescent intensities in the extravascular regions in the image 0 min after DEB injection in Figure 4g (Figure S10, Supporting Information). For L2 location, the *D*(10%) first increased for the initial 24 h and then decreased at a relatively slow speed while still covering a considerable area after 30 d's release (Figure 4i). After that, very few doxorubicin was detectable outside vessel. In addition, the *D*(50%) of DEB was much higher than that of EO‐DOX, suggesting comparatively slower decrease of extravascular drug concentration with the diffusion depth. Moreover, the drug diffusion depth decreased with the reduction of vessel diameter because of less microbeads in narrower vessels in DEB group. For instance, on Day 1, the *D*(50%) dropped from 1388 µm in L2 location with vascular diameter of 666 to 376 µm in L4 location with vascular diameter of 301 µm, which corresponds to an estimated quantity of beads of 37 in L2 and 7 in L4 assuming that the DEB microspheres were compacted inside the embolic vessels (Figure 4i; Figure S11, Supporting Information).

Furthermore, we built up an IVIVC based on the correlation between the doxorubicin concentration and corresponding grayscale (Figure 4j–l). Previously, Namur et al. quantitatively evaluated the extravascular drug concentration of DEB in patient liver explants based on the assumption of linear relationship of doxorubicin fluorescent signal with the drug concentration using a microspectrofluorimetry technique.^[^ ^50^ ^]^ We determined the ratio of the grayscale at diffusion depth of *n* µm to that adjacent to the vessel wall obtained from DLM model, i.e., *G_n_*/*G*_0_, as well as the ratio of the concentration at diffusion depth of *n* µm to that adjacent to the vessel wall derived from the human HCC samples, i.e., *C_n_*/*C*_0_ (Figure 4k; Figure S12, Supporting Information). The in vivo *C_n_*/*C*_0_ exhibited great linearity with the in vitro *G_n_*/*G*_0_ for various time points up to 30 d, confirming the effectiveness of the DLM model to mimic critical locoregional drug release environment of HCC (Figure 4l). The slope of the IVIVC curve was close to 1, which indicates that the DLM model produced comparable long‐term drug release kinetics of DEB as observed in human HCC. We tested the DEB in a rabbit VX2 liver cancer model and further confirmed the extravascular drug diffusion capability in the IVIVC curve (Figure S13, Supporting Information).

### 2.5 Quantification of Extravascular Doxorubicin Coverage

**Figure 5:**
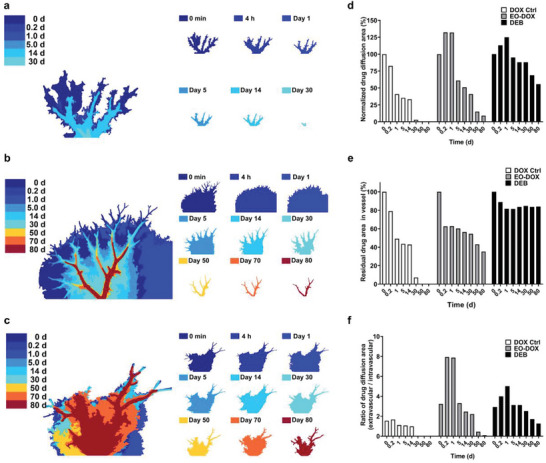
Comparison of locoregional doxorubicin coverage during drug release in DLM model. The change of drug diffusion areas of three drug formulations, i.e., a) DOX Ctrl, b) EO‐DOX, and c) DEB, up to 80 d are examined with diffusion regions of each time points fulfilled with distinctive colors. Both intravascular and extravascular doxorubicin coverages are quantitatively analyzed and compared, including d) normalized doxorubicin diffusion area, e) residual drug area in vessel, and f) ratio of extravascular drug covered area to corresponding intravascular drug occupied area.

To better depict the extravascular drug diffusion behavior, drug‐coverage regions at different time points are labeled in different colors (**Figure** **5**a–c). Unlike the DOX Ctrl showing continuous shrinkage of the extravascular drug diffusion region with time, both EO‐DOX and DEB groups exhibited a remarkable increase in the diffusion area until 24 h and then showed a gradual decrease at the latter time points. This indicates that the two embolic agents took about one day to reach the maximum drug release rate, which was balanced with a constant drug depletion rate in the DLM model. The drug coverage area of EO‐DOX even exceeded the observation view field at the early time points, but then quickly dropped to less than half of the maximum value on Day 5 (Figure 5d). By contrast, the DEB demonstrated much more steady drug diffusion region throughout the testing period up to 80 d (Figure 5d) and exceptional stability of intravascular drug coverage area with time (Figure 5e). Although EO‐DOX exhibited much larger drug coverage area than DEB within 24 h after endovascular embolization, which is mainly due to the instability of the emulsion causing large amount of drug instantaneously released into the surrounding tissues as well as systemic circulation, the severe initial drug burst of EO‐DOX was undesirable and could cause a high rate of adverse effects.^[^ ^8^ ^]^

We further determined the ratio of extravascular and intravascular diffusion area, which can roughly describe the drug diffusion capability (Figure 5f). Similarly, both embolic agents demonstrated superior diffusion capabilities to the DOX Ctrl, with the DEB exhibiting more controllability of the drug release pattern than EO‐DOX. At the last time point of 80 d, the ratio of extravascular to intravascular drug‐present area was still as high as 1.3 for the DEB group, whereas no drug was detectable for the EO‐DOX group, suggesting exceptional extended drug release behavior of DEB as compared to EO‐DOX (Figure 5f). On the other hand, both DEB and EO‐DOX demonstrated a strong dependence of the extravascular drug coverage on the dimension of the vessel branches, which is consistent with previous findings.^[^ ^42^ ^]^ This can be explained by the faster drug depletion rate from the comparatively smaller distal vessel branches providing much less volume to accommodate drug‐eluting embolic agents.

### 2.6 Quantification of Intravascular Doxorubicin Retention

**Figure 6:**
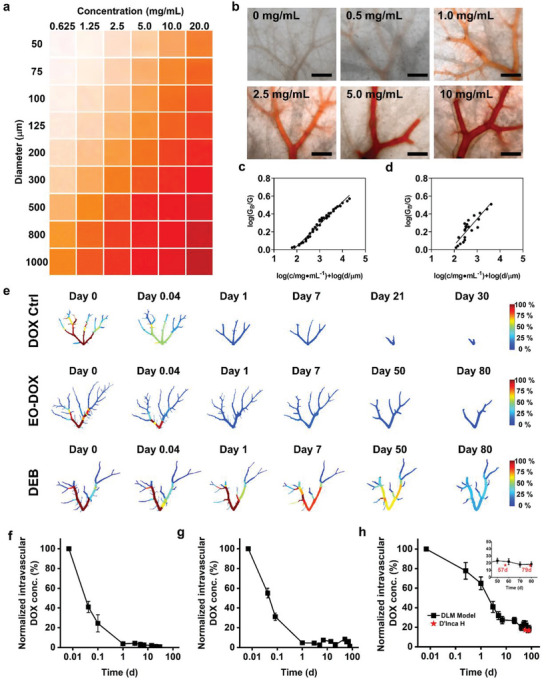
Quantification of intravascular doxorubicin concentration in DLM model. a) Bright‐field microarray images obtained from microfluidic channels with varying channel dimensions and drug concentrations. b) Bright‐field microscopy images of DLM model filled with doxorubicin solutions (0–10 mg mL^−1^) through the portal vein. Scale bar: 2 mm. c,d) Linear relationship of the light attenuation of doxorubicin solutions against log(*c*•*d*) is derived in both microfluidic channels (slope: 0.249; *R* ^2^: 0.969) and DLM model (slope: 0.293; *R* ^2^: 0.778). e) The concentration percentile maps of intravascular residual doxorubicin (branch level: P_1_–P_3_) at varying time points up to 80 d. f–h) Progression of residual doxorubicin concentration in the main vessel branches of the three embolic agents: DOX Ctrl (f), EO‐DOX (g), and DEB (h). Data are shown as means ± SEM (*n* = 10 replicates). The inlet figure in (h) highlights the comparable results of the residual drug concentration of in‐human HCC results reported by D'Inca et al.^[^ ^42^ ^]^

To further characterize the residual doxorubicin in vessel, a drug‐concentration quantitation method based on bright‐field microscopy images was developed (**Figure** **6**a–d). We designed a microfluidic‐based vascular model containing square channels with equal width and height varied from 50 to 1000 µm. Based on a concentration map obtained from the microfluidic bright‐field images (Figure 6a), an empirical equation modified from the Beer–Lambert law is derived

(1) $$A\equiv \log\;\frac{G_{B}}{G}=\varepsilon \log\left(c\cdot d\right)+k$$

where the light attenuation, *A*, is defined by the logarithm of the ratio of the grayscale (0–255) in the background, *G*_B_, to that in a specific site located within drug‐containing channels/vessels, *G*. *ε* is the light attenuation coefficient, and *c* is the drug concentration. *d* represents the optical path length, which can be the vascular diameter when we select a particular location in the center of the vessel. *k* denotes the light attenuation‐independent factor and may contribute to intensity loss related to light scattering. Clearly, the light attenuation of doxorubicin solutions increases with the drug concentration and the channel size (Figure 6a). Similar tendency is observed in the DLM model, where the images were obtained right after injection of the drug solution before significant diffusion happens (Figure 6b). A good linear relationship is established between the light attenuation and log(*c*•*d*) in both microfluidic model (Figure 6c) and DLM model (Figure 6d). No significant difference between the two fitted curves was observed based on the nonpair *t*‐test, and the fitted curve calculated by the microfluidic model was used for further measurement.

The concentration maps of residual doxorubicin in vessel branches of Levels P_1_–P_2_ at different time points were illustrated in Figure 6e. We found that the initial intravascular drug concentration decreased when getting into the vessel branches of smaller sizes, which can be attributed by the remarkable drug diffusion happened for both DOX Ctrl and EO‐DOX groups and comparatively low particle density for the DEB group. Note that, because of the difficulty to accurately quantify the drug concentration in small vessels (dia. < 100 µm), vessel branches of Levels P_3_–P_5_ were not included in the concentration map. The progression of the drug concentration in the main vessel branches of Level 1 close to the vessel root was plotted with time (Figure 6f–h). For DOX Ctrl group, the residual doxorubicin concentration in the main vessel branch quickly declined for 96.2% within 24 h and could hardly be detected after 30 d (Figure 6f). The EO‐DOX exhibited similar drug release behavior to DOX Ctrl for the first 24 h, showing slightly higher concentration in the main vessels close to the root only on Day 0.04 (Figure S14, Supporting Information). The remaining drug concentration of EO‐DOX group was only 4.7% in the main vessel branches after 24 h's release, which is close to the saturated drug concentration in the oil phase (i.e., 5.0%) (Figure 6g; Figure S7b, Supporting Information). Although most of the drug was released within 24 h for both groups, the extravascular drug diffusion depth after 24 h was dramatically enhanced in EO‐DOX as compared to DOX Ctrl. This confirms that, for the emulsion‐based embolic agent, the early fast release stage within 24 h was probably associated with gradual demulsification, while the latter extended‐release stage after 24 h could be attributed to the slow‐release kinetics from the oil phase.

Comparing with DOX Ctrl and EO‐DOX, the DEB group exhibited much more sustainable release kinetics due to the strong electrostatic interactions between the drug and DEB. More than 60% of doxorubicin was still left in the main vessel after 24 h's release. The remaining doxorubicin in vessels was 22.2% and 18.5% after 60 and 80 d, respectively. These results are comparable to the observations in resected liver specimens from patients (Figure 6h),^[^ ^42^ ^]^ which further confirm the validity of the DLM model at the latter drug release stage. Note that previous studies have been rarely reported in vivo results of either intravascular drug retention and extravascular drug diffusion of the emulsion‐based embolic agents such as EO‐DOX probably due to the liquid nature of the emulsion that renders difficulty to handle histological sections without disruption of the embolics. In this regard, the DLM model provides an ideal platform to characterize the drug release behavior of liquid‐based drug depots in a nondestructive manner.

To compare, we further tested the drug release behavior of DEB in DLM model using perfusion solution of 0.9% NaCl instead of pure water, which exhibits dramatically accelerated drug release rate with less than 1% of drug remaining in the main vessel branches after 7 d's release (Figure S15, Supporting Information). Therefore, the drug release performance of DEB is strongly affected by the perfusion solution since the positively charged sodium ions can gradually break down the electrostatic interactions between positively charged doxorubicin with the negatively charged DEB.^[^ ^51^ ^]^ The appropriate drug release rate achieved by perfusion with pure water but not 0.9% NaCl indicates minimal cellular activities and blood drainage in the embolized tumor environment. In addition, the drug depletion speed can be facilely controlled in the DLM model, showing flexibility of the in vitro model to adjust drug release kinetics for different conditions when needed. To compare, we tested the drug release behavior of DOX Ctrl, EO‐DOX, and DEB in dialysis bags using PBS buffer (pH = 7.4). Both EO‐DOX and DEB exhibited an initial drug burst within 12 h but the remaining drug could be barely released from the dialysis bags at the latter time points, which can hardly provide any meaningful information of the long‐term intratumoral drug release behavior as observed in the DLM model (Figure S16, Supporting Information).

### 2.7 Drug–ECM Interactions in DLM Model

**Figure 7:**
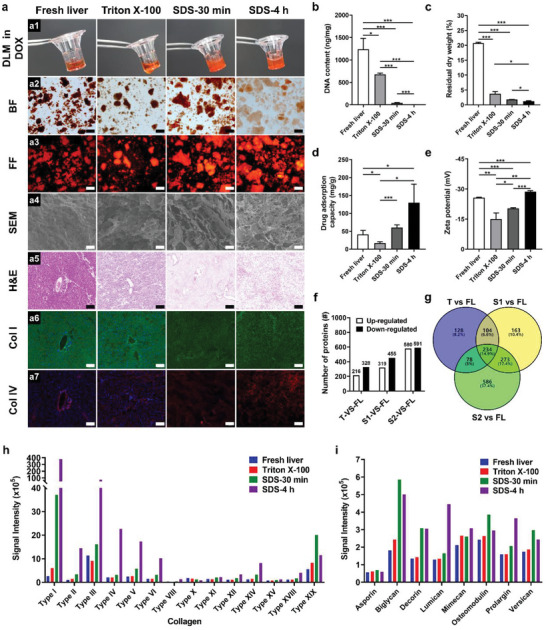
Evaluation of ECM compositions and drug–ECM interactions in fresh and decellularized livers. a) Characterization of rat livers pre‐, during, and after decellularization process. The fresh liver (FL) was first perfused with Triton X‐100 for 30 min (Triton X‐100, T), and then perfused with SDS solution for 30 min (SDS‐30 min, S1) or 4 h to finally derive the DLM model (SDS‐4 h, S2). The cryogenically pulverized liver samples were incubated with doxorubicin solution for 24 h (a1), showing strong drug adsorption capacities (a2: bright‐field image; a3: fluorescent image). SEM images of the liver samples demonstrate increasing porosity with the decellularization process (a4). Liver sections were further stained with hematoxylin–eosin (H&E, a5), or immuno‐stained for type I collagen (Col I, a6) and type IV collagen (Col IV, a7). Sections were counterstained with DAPI (blue). Scale bars: 200 µm (a2,a3), 20 µm (a4), and 100 µm (a5–a7). Quantitative evaluations of the liver samples were performed, including measurements of b) DNA content, c) residual dry weight, d) drug adsorption capacity, and e) zeta potential. Data are shown as means ± SD (*n* = 3 replicates). f–i) ECM protein compositions were analyzed by mass spectrometry‐based proteomics. Venn diagrams of differentially expressed proteins detected in decellularized liver samples as compared to the fresh liver group (f). The overlap represents differentially expressed proteins that are common in livers after decellularization. The number of differential expression proteins of decellularized livers comparing with fresh liver (g). Collagens (h) and proteoglycans (i) identified by proteomic analyses of fresh and decellularized liver tissues.

Electrostatic interactions between the drug and the tissue play a crucial role in drug transport, uptake, and retention.^[^ ^52^ ^]^ The chemotherapeutic drug of doxorubicin is known for its electropositive nature.^[^ ^9^, ^46^ ^]^ The electronegativity of tissues mainly comes from extracellular anionic proteoglycans containing high negative fixed charge density (FCD) as well as negatively charged cells.^[^ ^53^ ^]^ To investigate if the decellularized organ model could provide biomimicking electronegative environment for the locoregional drug release test, we first tested the drug adsorption capacity of the liver models with varying degree of decellularization by incubating the liver pieces with doxorubicin solution (**Figure** **7**a1). We found that all liver samples exhibited strong drug adsorption in both bright‐field and fluorescent images (Figure 7a2–a3). The SEM and histological images confirm the efficient decellularization process that gradually removed the nuclei and produced acellular tissues with highly porous morphologies (Figure 7a4–a7), which is consistent with the observations in the continuous reduction of DNA content (Figure 7b) and residual dry weight (Figure 7c).

More specifically, compared to fresh liver, the drug adsorption capacity first decreased with partial decellularization by triton X‐100 due to the removal of cellular components, but then remarkably increased for the SDS‐treated liver samples owing to the enrichment of the ECM (Figure 7d). Correspondingly, the zeta potential presented an initial change from −27 to −15 mV, and then dropped down to −29 mV with the decellularization process (Figure 7e). This suggests that the DLM model demonstrates comparable electronegativity with the fresh liver. Note that the drug adsorption capacity of the DLM model is especially enhanced, which may be explained by the porous structures of the enriched ECM providing high interfacial surface for the drug adsorption. Such decellularized matrix itself holds great potential to serve as an efficient drug carrier due to the exceptional loading capacity with positively charged pharmaceuticals.

We further performed mass spectrometry‐based proteomic analyses to interpret the effect of decellularization processing on the tissue composition of the liver samples. In the unfractionated and label‐free proteomic tests, total 1 096 186 spectra and 26 260 identified peptides including 24 745 unique peptides were obtained, which resulted in 5358 proteins (false discovery rate ≤ 1%.). A total of 1566 differentially expressed proteins (DEPs) were identified with a citation of “Fold change > 1.2 and *Q*‐value < 0.05”. Both enriched and depleted proteins increase with the decellularization process (Figure 7f) with 234 common DEPs detected across the three samples (Figure 7g). Among the 580 enriched and 591 depleted DEPs for DLM model as compared to fresh liver (i.e., S2 vs FL), 50 identified proteins are associated with extracellular structures based on eukaryotic orthologous group database (Figure S17, Supporting Information), showing enrichment of 96% of the ECM proteins in the DLM (Figure S18 and Table S7, Supporting Information).

Furthermore, we found that the collagen proteins, especially the fibrillar collagens such as Types I, II, III, VI collagens, were dramatically enriched during the decellularization processing (Figure 7h; Table S8, Supporting Information). Proteoglycans are also well retained, especially for biglycan, decorin, lumican, and prolargin, during the decellularization process, but in a much less degree than the collagens (Figure 7i; Table S8, Supporting Information). Similar results of both collagens and proteoglycans were obtained in duplicated experiments (Figure S19, Supporting Information). Unlike the collagen group showing continuous increasing trend with decellularization, the abundance of most proteoglycans first increases when treated through triton X‐100 for 3 h and SDS for 30 min, and then decreases with further SDS treatment. This indicates that the proteoglycans may be partially depleted at the latter stage of decellularization, but the remaining enriched proteoglycans with high negative FCD (e.g., biglycan carrying with chondroitin sulfate chains, decorin with dermatan sulfate chains, lumican with keratan sulfate chains)^[^ ^54^, ^55^ ^]^ still contributes to the low zeta potential in the DLM model. Therefore, the DLM model presents a powerful template to replicate the intratumoral electronegative environment for the balance of drug diffusion and depletion.

## 3 Discussion

In this work, we presented a strategy to engineer a 3D tumor‐mimicking drug release model combined with deep learning‐based computational analyses that for the first time enabled quantitative evaluation of the spatiotemporal drug release behaviors within tumor tissues. The deficiency of appropriate in vitro models to probe intratumoral pharmaceutical kinetics is a versatile problem for evaluating translational medicines associated with complex treatment modalities. Our previous work has shown that the acellular model could provide an optical translucent scaffold for direct chemoembolization examinations.^[^ ^28^, ^56^, ^57^, ^58^ ^]^ Here the engineered drug release model successfully circumvented the limitations of current in vitro models through employing decellularized liver organ with the following three key features for locoregional drug release characterizations. Specifically, the intact vasculature systems obtained from native organ provide mature vessel branches with dimensions close to those in human HCC, which allows the direct characterization of clinical‐relevant embolic agents. Second, the acellular ECM offers a biomimetic electronegative scaffold for extravascular drug diffusion, which is especially crucial for testing non‐neutral drug agents such as the widely applied anticancer drug, doxorubicin. Third, although no cellular components are available in the decellularized model, the tumor‐specific drug depletion can be achieved through facilely tuning the buffer perfusion throughout the drug testing platform to reproduce the effects of cellular uptake, metabolism, and potential drainage of drugs in tumor. Additionally, the DLM‐based drug testing platform exhibits superior mechanical stability for long‐term nondestructive microscopy evaluations up to 80 d.

Furthermore, using deep learning‐based computational analyses, we for the first time extended the computational research tool to drug release‐associated vessel segmentation and skeleton extraction, which plays a vital role in the quantitative assessment of spatiotemporal drug release kinetics. We proposed a DAU‐GAN‐based deep learning pipeline and obtained significantly enhanced accuracy for drug‐containing vessel segmentation, despite of the difficulty to differentiate the drug‐containing vessels with fuzzy vessel boundaries during the drug release process. The root and branch level classification system designed in the work is particularly useful for quantitative assessment of the complex vascular network. In addition, the empirical correlation of the intravascular drug concentration with image‐based information for the first time enables the creation of drug concentration mapping at the tumor site, greatly enhanced the derivation of the spatial information of intravascular drug distribution.

Building an effective IVIVC is of paramount importance to verify if in vitro models are appropriate for in vivo predictions. For TACE treatment, current efforts of IVIVC modeling mainly focus on the short‐term correlation with systematically released drug within 24 h upon clinical operation and heavily reply on in vivo pharmacokinetic data obtained from plasma concentrations.^[^ ^59^, ^60^, ^61^ ^]^ Although tremendous attention have been driven to the investigation of embolic agents with optimized properties,^[^ ^62^, ^63^, ^64^ ^]^ no model has been able to efficiently compare their intratumoral responses side by side.^[^ ^10^, ^17^ ^]^ Here we successfully established a linear IVIVC of DEB with in‐human results based on extravascular drug diffusion performance within one month, and further verified the correlation of intravascular drug retention at the late stage of drug release up to 80 d.

Using the DLM model, we achieved comprehensive comparison of the two most used types of embolic agents, i.e., liquid embolic emulsion of EO‐DOX and microparticle‐based embolic agent of DEB, which may provide valuable insights for the long‐lasting debate of these embolotherapies. We found that, compared to EO‐DOX, DEB demonstrated exceptional drug release behaviors in terms of sustained intravascular drug retention and enhanced extravascular drug diffusion over 80 d throughout the observation time period. Nevertheless, the initial drug release rate of DEB was still very fast and quickly dropped with time, exhibiting over 70% of the drug released within 7 d but less than 10% of the drug depleted within the next 70 d. After 80 d's release, nearly 20% of the doxorubicin remained in the DEB showing an extremely slow release rate, which can be explained by the tight electrostatic interactions of the positively charged drug with the negatively charged microspheres. For cTACE, the EO‐DOX showed a burst release of 95% of the drug within 1 d, resulting in a huge drug diffusion area outside the blood vessels in the initial stage. What is unexpected, even if there is very little drug left in EO‐DOX after 1 d, is the remarkable sustained release effect of the remaining drug dissolved in the oil phase. The only 5% remaining drug in EO‐DOX demonstrated a greatly slowed release rate with a notable extravascular drug diffusion capability over 30 d. Accordingly, except for the first day's burst release, the cTACE exhibited an overall extravascular drug diffusion performance only slightly inferior over that of DEB‐TACE within 30 d's release time period. Therefore, both types of embolic agents may be improved in different ways. Note that both EO‐DOX and DEB used in TACE treatments are non‐degradable and permanently retained inside the tumor vessel. For DEB‐TACE, the locoregional drug release performance of DEB may be enhanced by incorporating additional driven force to trigger drug release, e.g., the endorsement of appropriate degradability, at the latter release stage. For cTACE, the development of better oil phase with improved drug‐loading properties may significantly enhance the chemotherapeutic effect of the embolic agent.

We believe that the soundness of the 3D drug release model with effective IVIVC, which proves the reliability to compare various types of clinical‐relevant agents, shows the promise for use as a powerful research tool to facilitate the development and optimization of translational drug compositions while leveraging comprehensive information of the whole drug release process from minimum organ samples for various locoregional treatments besides the TACE treatment. Further endeavors may also extend to developing tumor‐bearing decellularized organ models to provide a better tumor‐mimicking template for pharmaceutical testing.

## 4 Experimental Section

### Fabrication of Decellularized Liver Organ

Decellularized rat liver organ was prepared according to a modified protocol in the previous work.^[^ ^28^, ^65^ ^]^ All animal experiments were approved by the Institutional Animal Care and Use Committee at the Southern University of Science and Technology (SUSTC‐JY2018034). Healthy male Sprague Dawley rats (8–9 weeks old) were euthanized by carbon dioxide gas asphyxiation and underwent a laparotomy procedure to harvest the liver organ with both hepatic vein and portal vein cannulated with flat pinhead needles. The harvested liver was stored at −80 °C before decellularization. For decellularization, the liver was first thawed and then perfused with deionized water for 30 min, 4% Triton X‐100 containing 0.02% EGTA for 3 h, 1% SDS solution for 4 h to eliminate cellular components. The perfusion process was performed through the portal vein at a flow rate of 3.0 mL min^−1^. The decellularized liver was fixed at 4% paraformaldehyde for 24 h and perfused with deionized water for 30 min to remove residual chemicals before use.

### Chemoembolization Test in DLM Model

Two typical embolic agents, i.e., EO‐DOX and doxorubicin‐loaded DEB, as well as a DOX Ctrl were chosen for chemoembolization test in the DLM model. EO‐DOX was derived by mixing aqueous doxorubicin solution (5.0 mg mL^−1^) with ethiodised oil (Lipiodol Ultra Fluide, Guerbet) using a three‐way stopcock at a volume ratio of 1:2. DEB was prepared by loading 2 mL of 25.0 mg mL^−1^ doxorubicin solution into 2 mL DC Bead M1 microspheres (caliber 70–150 µm, Biocompatibles, UK). In the paper of Namur et al. for IVIVC establishment, the DEB applied were from the same company with similar sizes (DC Bead, Biocompatibles UK, caliber 100–300 µm, 37.5 mg doxorubicin mL^−1^ bead).^[^ ^50^ ^]^ They obtained in‐human results from patient liver explants at varying time points from 8 h to 36 d. DOX Ctrl contains 5.0 mg mL^−1^ of doxorubicin solution. In the DLM model, the embolic agents were selectively injected into one main portal vein branch of the left lateral lobe. Distilled water was then applied to continuously perfuse throughout the hepatic vein at 1.0 mL min^−1^ unless otherwise specified. Stereomicroscopy (ZEISS SteREO Discovery V12) was employed to obtain bright‐field and fluorescent images of the DLM model to monitor chemoembolization performance at predetermined time points up to 80 d. For the fluorescence detection, doxorubicin was imaged using the Cy3 filter (530–560 nm excitation/573–647 nm emission).

### Image Preprocessing for Deep Learning Pipeline

Considering that supervised learning could typically deliver more accurate segmentation, 55 ground truth vessel segmentation labels were manually created by four experts. The four experts were familiar with the experimental images. Two of them used a Procreate App with an Apple Pencil to manually mark the drug‐contained vessels as labels on an iPad (Figure S20, Supporting Information). The other two experts modified the marked labels if necessary, ensuring the correctness and consistency of the finally annotated ground truth. Data augmentation, including flipping, rotating, was conducted at every 4° in the range of (4°, 360°), and randomly adjusting the contrast and brightness, for each of the 55 images.

### Deep Learning Network Architecture

A DAU‐GAN was adopted for segmentation of drug‐containing vessels. The GAN contains a generator (G) and a discriminator (D), which are trained alternatively to make the predicted output indistinguishable from the ground truth.^[^ ^66^ ^]^ Specifically, G is trained to generate fake vessels that are as similar to the ground truth as possible, and D is trained to distinguish the real label from the fake one. G adopts an encoding–decoding U‐Net structure and complements an attention module on each Skip Connection path to endow excellent generation capability. In detail, the Conv Block has a convolution‐BatchNorm‐ReLu structure, and the number in the block represents the number of the outputted feature map channels. The MaxPool (Conv.Trans) performs downsampling (upsampling). The encoder–decoder pair is concatenated via the Skip Connection path with DA.^[^ ^67^ ^]^ The fake image is generated through 1 × 1 convolution and sigmoid. D simply consists of multiple stacked Conv Blocks. Adam optimizer with an initial learning rate of 10^−4^ was used to train the DAU‐GAN. Fourfold cross‐validation analysis was performed. Both binary segmentation and skeleton extraction results were validated quantitatively and qualitatively. Overall, the automatically obtained segmentation results had an average DSC of 88.4%, indicating high segmentation accuracy. Neither any large segmentation error nor any failure was observed. It was observed that the subsequent skeleton extraction and topology analyses were very sensitive to the segmentation accuracy, so the automatically predicted segmentation results were manually corrected when necessary. Figure S21 of the Supporting Information demonstrates manual correction of a root node (i.e., the starting point of the vascular branches), which was performed when the targeted drug‐containing vessels were not fully incorporated.

### Vessel Skeleton Extraction

For the binary vessel segmentation, an automatic skeleton extraction algorithm was employed. The algorithm iteratively removes redundant pixels from the vascular outer edge until there leaves with only a connected skeleton of a single‐pixel width.^[^ ^27^ ^]^ The vessel skeleton obtained represents the extent of the drug diffusion process. To be specific, the algorithm designs deletion rules for each vascular pixel's eight adjacent pixels, which consists of two processes; one process removes redundant pixels in the north‐west corner and the south‐east boundary, and the other one removes redundant pixels in the south‐east corner and the north‐west boundary. The final remaining matrix is defined as the skeleton matrix, where 1 (0) denoted the skeleton pixel (background).

### Skeleton Branch Classification

A customized algorithm was designed to automatically divide the entire skeleton into five levels according to the length of the vascular branches and the order of bifurcation. Figure S2 of the Supporting Information illustrates three representative examples of the skeleton branch classification results respectively from DOX Ctrl, EO‐DOX, and DEB. The nonzero skeleton pixels in the skeleton matrix are defined as the root node, edges, bifurcation nodes, and leaf nodes. The root node represents the starting point of the vascular branches. The bifurcation nodes are located at the intersections of different vascular branches, and the leaf nodes end the vascular branches. The edge denotes the branch section between each two nearby bifurcation nodes or between the bifurcation node and its closest leaf node. The number of edge pixels is added up to quantify the length of the vascular branches. To eliminate the impact of slight displacements during the acquisition of longitudinal images of the same sample, the algorithm automatically detects the first main bifurcation point of the vascular root and takes it as P_1_ level's root node (Root). To determine the Root, three bifurcation nodes with the top three diameters are first selected as candidate Roots. Since some small vessel branches near the real Root also have large diameters, the candidate Root is chosen farthest from the edge of the image as the real Root. After identifying the Root, the number of main branches, *n*, that extends from the Root, is determined and the shortest path between the Root and each leaf node using the Dijkstra algorithm is calculated.^[^ ^68^ ^]^ The “*n*” leaf nodes that extend through the main branches with the farthest distance from the Root are defined as the P_1_ level nodes, and the paths from the Root to the P_1_ level nodes are defined as the P_1_ level paths. Then, all bifurcation nodes on the P_1_ level paths serve as the P_2_ level root nodes. In the same way, the algorithm starts from the P_2_ level root nodes to localize the corresponding P_2_ level nodes and paths. Through multiple iterations, all the leaf nodes and the corresponding paths are divided into different levels. All leaf nodes above the P_5_ level are classified as P_5_ level as well. In this way, the vascular topology was successfully established.

### Topological Quantification

Through the aforementioned vascular segmentation and topological analysis pipeline, various indicators that are critical to the drug diffusion process can be automatically determined, including the number, length, and diameter of vessel branches at different levels. Specifically, vascular diameter is obtained from the binary segmentation result and the corresponding skeleton matrix. The distance from each skeleton pixel to the boundary of the vessel (white area) is calculated, wherein the minimum distance that gets doubled is defined as the diameter of the vessel for that specific skeleton pixel. The average diameter along the skeleton path can then be calculated for vessel branches.

### Extravascular Drug Diffusion Characterization and IVIVC

Extravascular drug diffusion behavior was evaluated based on fluorescent microscopy images of DLM model obtained at predetermined time points. In the DLM model, the overall drug diffusion region was derived by selecting the area from the fluorescent images with grayscale from 1 to 255 (dark: 0; white: 255) using ImageJ software (NIH, Bethesda, MD). For each drug formulation group, four vascular locations along with one main Level P_1_’s branch (L1: 800–1000 µm; L2: 600–800 µm; L3: 400–600 µm; and L4: 200–400 µm) were selected to analyze extravascular drug diffusion capacity. For the DEB group, IVIVC was established between the extravascular drug diffusion behavior of L2 location of DLM model with the in‐human results reported by Namur et al.^[^ ^50^ ^]^ The data of extravascular drug concentration at different diffusion depth and time points in human HCC were extracted from Figure 1A in the paper of Namur et al.^[^ ^50^ ^]^

### Spatiotemporal Drug Release Mapping and Quantification

To establish the correlation of intravascular doxorubicin concentration with the diameter of the vessel and the image grayscale, a PDMS‐based microfluidic vascular model comprising straight square channels with equal width and height varied from 50 to 1000 µm was designed. A series of doxorubicin aqueous solutions with concentration varied from 0.625 to 20.0 mg mL^−1^ were injected into the microfluidic channels for bright‐field microscopy imaging. Based on the drug concentration, vessel diameter and grayscale, an empirical equation modified from the Beer–Lambert law was derived. The empirical equation was further confirmed in the DLM model by injecting drug solutions into the portal vein and instantaneously captured the bright‐field images before significant drug diffusion happened.

To derive spatiotemporal drug release mapping of different embolic agents in the DLM model, the empirical equation modified from the Beer–Lambert law, Equation (1), is reformulated as

(2) $$\log\frac{G_{t}}{G_{s}}=\varepsilon \left(\log\frac{c_{s}}{c_{t}}+\log\frac{d_{s}}{d_{t}}\right)$$

The first edges (Figure S2, Supporting Information) extended from the Root are defined as the source edges of the Root, and then the average image grayscale and the average diameter of all source edges as *G*_s_, *d*_s_ are calculated. The drug concentration is assumed in source edges, *c*_s_, equals to the original drug concentration in the prepared embolic agents, *c*_0_, at the zero‐time point. Along each‐level vessels’ paths, the edges were defined as the target edges, and the corresponding target edges’ average image grayscale *G*_t_ and diameter *d*_t_ were automatically calculated. By experiment, *ε* = 0.249 was obtained. With the above parameters, each target edge’ concentration *c*_t_ along the vascular path can be figured out. To reduce the influence of noise, an edge as the smallest unit was taken when estimating the drug concentration degree throughout the vessel skeleton. Moreover, the local drug concentration of the P_1_–P_2_ level vessels was only quantified, considering that the distal small vessels are seriously affected by noise and the vascular trunk is sufficient to show the overall trends in terms of drug distribution.

### Characterization of Decellularized Scaffolds

To evaluate the influence of decellularization process on hepatic scaffold, four liver samples that were decellularized into different degrees were tested. For the Triton X‐100 group (group T), the decellularization processes were stopped after finished perfusion of Triton X‐100. Besides, livers were also collected as another two groups (groups S1, S2) after decellularization with SDS for 30 min and 4 h, respectively. The fresh liver was used as a control group (group FL). Finally, livers were washed thoroughly with distilled water and fixed for histology and SEM or frozen at −80 °C until mass spectrometry and dsDNA analyses. For histological analysis, the liver samples were embedded in paraffin and processed for staining with hematoxylin and eosin. Besides, immunohistochemical staining was undertaken using antibodies for Collagen I (Servicebio) and Collagen IV (Servicebio). Nucleic acid was counterstained by DAPI (Servicebio). The images were captured with Nikon Eclipse C1 fluorescent microscope. In addition, the morphologies of fresh and decellularized livers were examined by a field emission scanning electron microscope (Merlin SEM, Zeiss, Germany). Samples prepared for SEM were dehydrated with a series of ethanol solutions for 15–20 min separately (50%, 70%, 90%, 100%), precooled at −80 °C for 4 h, and then dried by lyophilization for 24 h. After sputter‐coated with a 10 nm layer of Pt, SEM images were captured at an acceleration voltage of 7 kV. For DNA analysis, these liver samples were lyophilized and cryogenically pulverized under liquid nitrogen. DNA extraction from the liver tissues was performed using a TaKaRa MiniBEST Universal Genomic DNA Extraction Kit (Takara, 9765, Japan). Double strain DNA (dsDNA) was quantified with Qubit dsDNA HS assay kit (Invitrogen, USA) according to the manufacturer's instructions. Relative residual dry weight of decellularized livers was determined by measuring the dry weight of the liver samples upon freeze‐drying for 4 d as compared to the weight of the fresh liver before decellularization. The measurements of the zeta‐potential of the DLM particles were carried out on a Malvern Zetasizer Nano analyzer at 25 °C (Malvern Instruments Ltd., Malvern, Worcestershire, UK). Micron‐sized DLM samples were suspended in 1.0 mL deionized water and vortexed for 2 min before measurement.

### Drug Adsorption Capability

The drug adsorption capability of fresh and decellularized livers was measured by incubating with doxorubicin solutions in 24‐well transwell plates. Specifically, dried powders of liver samples (≈5 mg) placed in the transwell inserts (0.8 µm, Corning Co., Corning, NY, USA) were incubated with 1.5 mL of 250 µg mL^−1^ doxorubicin solutions at 37 °C for 24 h until the tissues were saturated with doxorubicin. The tissue powders were observed under microscope while the doxorubicin in the compartment was tested using high performance liquid chromatography (HPLC) system (Waters, Milford, MA, USA) equipped with a Waters Sunfire C18 analytical column (5 µm, 4.6 × 150 mm) and a UV/vis detector set at 254 nm. The drug samples were mixed with acetonitrile (1:1 v/v) to precipitate soluble proteins, and then centrifuged at 12 000 rpm for 15 min. The collected supernatant was diluted 500 times by mobile phase for HPLC characterization. The drug adsorption capability of liver tissues was calculated by the decrement of doxorubicin concentration in the compartment.

### Proteomics Analyses

To examine the enrichment of extracellular protein compositions during decellularization, four liver samples (FL, T, S1, S2) were tested for proteomics analyses. Proteins were extracted from the tissue samples using lysis buffer (7 mol L^−1^ urea, 2 mol L^−1^ thiourea, 20 mmol L^−1^ Tris‐HCl, PH = 8.0) with phosphatase inhibitor cocktail (Roche), reduced with 10 mmol L^−1^ dithiothreitol, and alkylated with 55 mmol L^−1^ iodoacetamide. Proteolysis of extracted proteins (100 *µ*g for each sample) was performed with trypsin solution (trypsin:protein = 1:20, w/w) at 37 °C for 4 h. Eightplex iTRAQ reagents (iTRAQ kit, Cat No. 4375249C, AB Sciex, Framingham, MA) were employed to label the digested peptides, i.e., 113 (FL), 114 (T), 115 (S1), and 119 (S2), respectively. The labeled peptides were separated into twenty fractions using a liquid phase system (Shimadzu LC‐20AB, Tokyo, Japan) equipped with a 5 µm 4.6 × 250 mm Gemini C18 column.

Using an HPLC system (Thermo UltiMate 3000 UHPLC), the peptide fractions were enriched in a trap column, desalted, and then separated through a self‐packed C18 column (75 µm internal diameter, 3 µm particle size, 25 cm column length) at a flow rate of 300 nL min^−1^. Two mobile phases were used, i.e., mobile phase A (2% acetonitrile, 0.1% formic acid) and mobile phase B (98% acetonitrile, 0.1% formic acid). The gradient parameters were set as 0–5 min with 5% mobile phase B, 5–45 min with mobile phase B linearly increased from 5% to 25%, 45–50 min with mobile phase B from 25% to 35%, 50–52 min with mobile phase B from 35% to 80%, 52–54 min with 80% mobile phase B, and 54–60 min with 5% mobile phase B. The separated peptides were ionized by a nanoESI source and then processed into a tandem mass spectrometer (Q Exactive HF‐X MS, Thermo Fisher Scientific, San Jose, CA) using a data‐dependent acquisition mode. Specifically, scanning was performed using an ion source voltage of 1900 V with MS1 scanning range set at 350–1500 m/z (resolution: 60 000) and MS2 starting m/z fixed at 100 (resolution: 15 000). For MS2 fragmentation, the ion screening conditions were set with the charge from 2+ to 6+, and the top 20 parent ions showing a peak intensity exceeding 10 000. The fragment ions were detected in Orbitrap mass analyzer (Thermo Fisher Scientific, San Jose, CA). The automatic gain controls for MS1 and MS2 were set to 3 × 10^6^ and 1 × 10^5^, respectively.

Proteins detected from the tandem mass spectra were identified and quantified using IQuant software (BGI, Shenzhen, China) that integrates Mascot Percolator algorithm. The acquired data were searched against the reference proteome of *Rattus Norvegicus* based on the UniProt Database (https://www.uniprot.org/). Totally 1 096 186 spectrums were generated, 26 260 peptides and 5358 proteins were identified with a false discovery rate ≤ 1%. DEPs were determined by a threshold of fold change > 1.2 and *Q*‐value < 0.05 in single replicate.

### Statistical Analyses

Data analyses were performed using GraphPad Prism 8 (GraphPad Software Inc., San Diego, CA, USA). For evaluation of deep learning‐based vessel segmentation and skeleton extraction, two‐sided paired *t*‐test was performed to assess the difference of DSC and ASSD. For evaluation of ECM compositions and drug–DCM interactions, two‐sided unpaired *t*‐test was performed to compare the liver samples. *p*‐value of < 0.05 was considered statistically significant, i.e., *P < 0.05, **P < 0.01, and ***P < 0.001.

## Conflict of Interest

The authors declare no conflict of interest.

## Supporting information

Supporting Information

## Data Availability

The data that support the findings of this study are available from the corresponding author upon reasonable request.
