# Supplementary material for: A 3D Tumor‐Mimicking In Vitro Drug Release Model of Locoregional Chemoembolization Using Deep Learning‐Based Quantitative Analyses
Source: Adv Sci (Weinh). 2023 Feb 15;10(11):2206195. doi: 10.1002/advs.202206195 (PMC10104640; doi:10.1002/advs.202206195)
Supplement: Supplementary file 1 — Supporting Information [file ADVS-10-2206195-s001.pdf]

ADVANCED SCIENCE
Open Access

Supporting Information

for *Adv. Sci.*, DOI 10.1002/advs.202206195

A 3D Tumor-Mimicking In Vitro Drug Release Model of Locoregional Chemoembolization Using Deep Learning-Based Quantitative Analyses

Xiaoya Liu, Xueying Wang, Yucheng Luo, Meijuan Wang, Zijian Chen, Xiaoyu Han, Sijia Zhou, Jiahao Wang, Jian Kong, Hanry Yu, Xiaobo Wang, Xiaoying Tang and Qiongyu Guo**

Supplementary Data

A 3D tumor-mimicking in vitro drug release model of locoregional chemoembolization using deep learning-based quantitative analyses

Xiaoya Liu,[#] Xueying Wang,[#] Yucheng Luo, Meijuan Wang, Zijian Chen, Xiaoyu Han, Sijia Zhou, Jiahao Wang, Jian Kong, Hanry Yu, Xiaobo Wang, Xiaoying Tang,[*] and Qiongyu Guo[*]

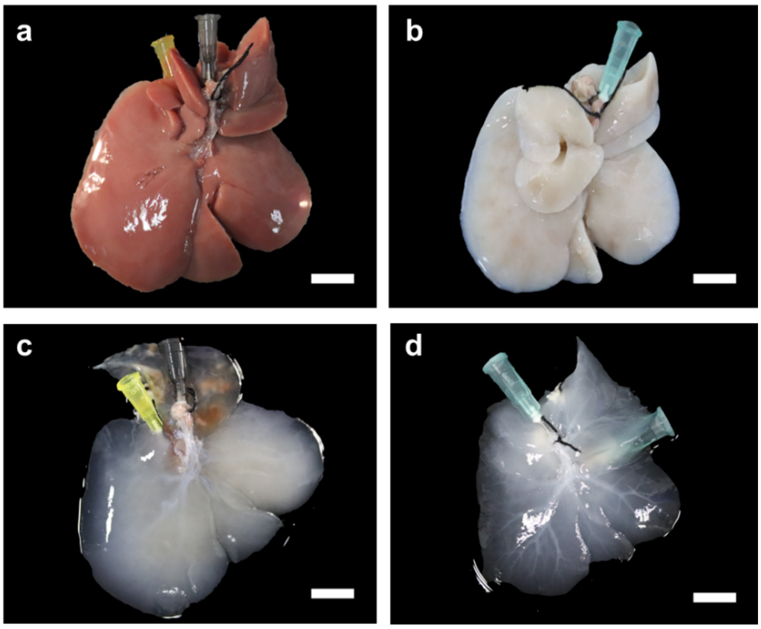

Fig. S1. Representative images of rat livers during decellularization process. The fresh liver (**a**) was first perfused with Triton X-100 solution for 3 h (**b**), and then perfused with SDS solution (**c**, 30 min; **d**, 4 h) to derive the acellular liver model for drug release test. Scale bar: 1 cm.

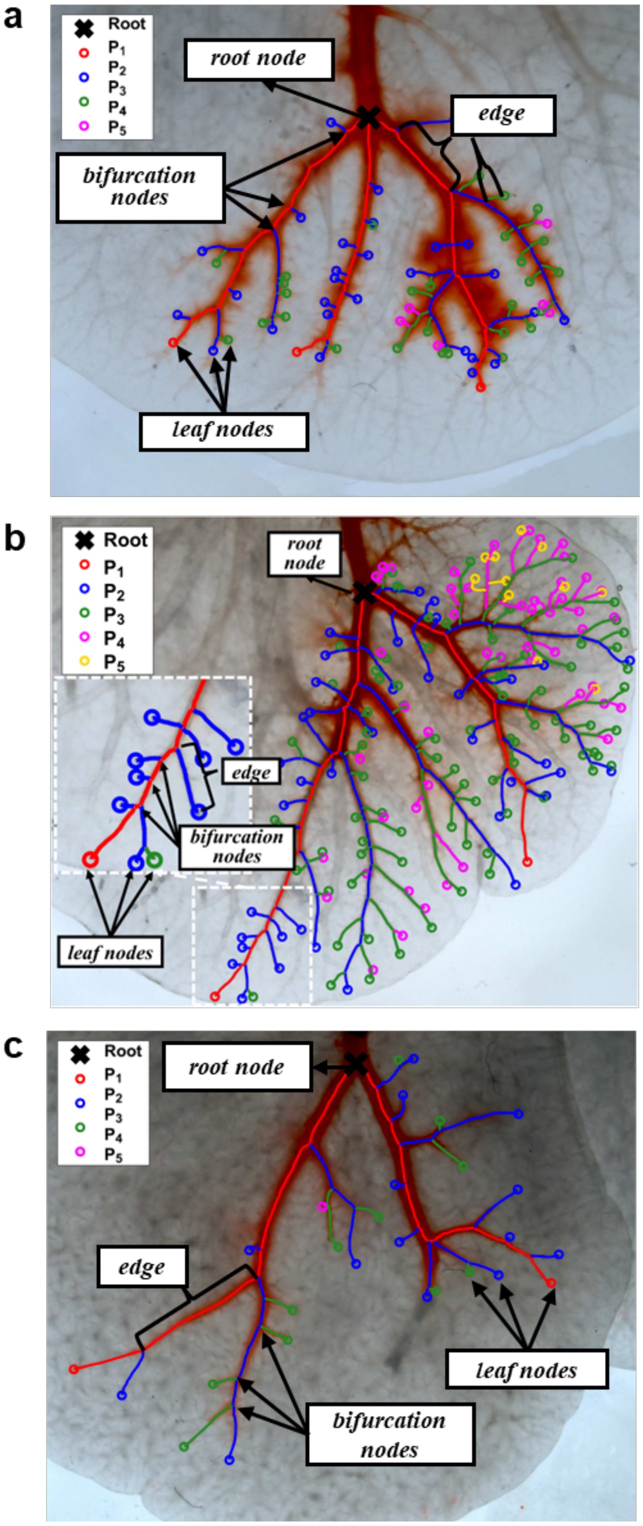

Fig. S2. Illustration of skeleton extraction and classification of drug-containing vessels: DOX Ctrl (**a**), EO-DOX (**b**) and DEB (**c**)**.** The vessel skeleton is defined as 4 elements: root node, bifurcation nodes, leaf nodes, and edge. The vessel branches are divided into 5 levels with a custom-designed rule, which is marked in different colors.

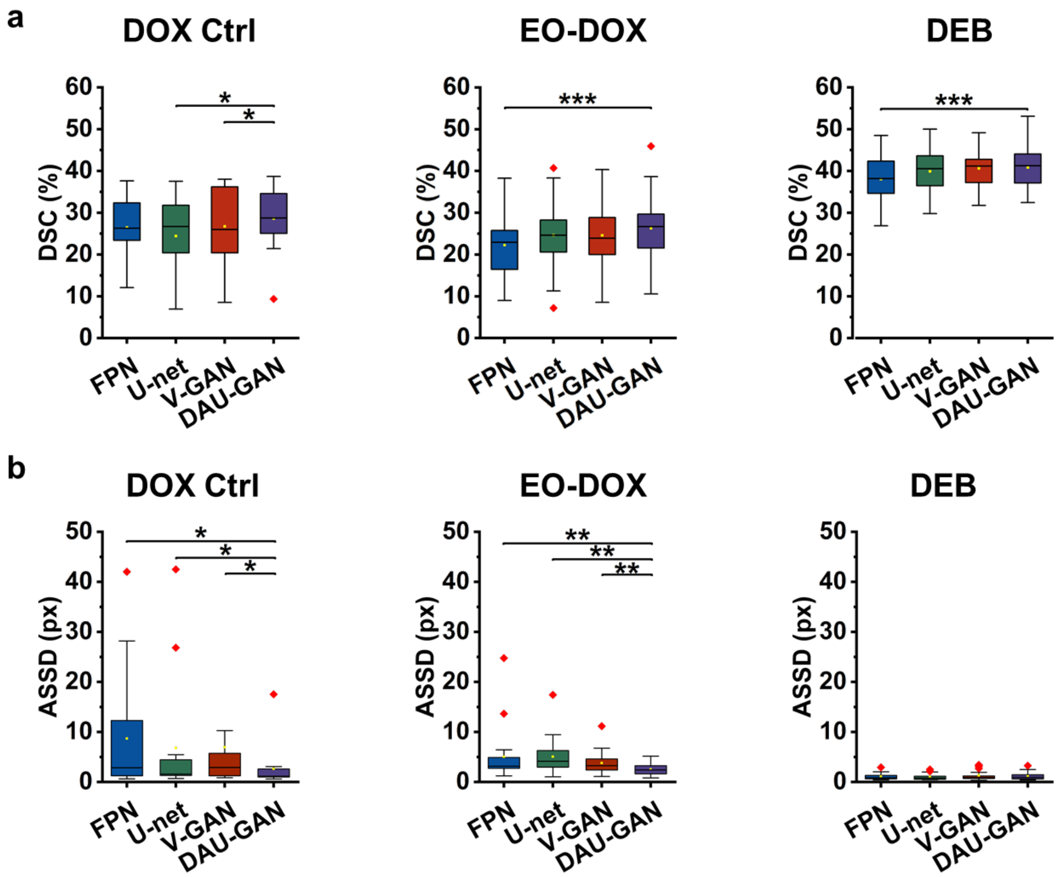

Fig. S3. Box plots of DSC (a) and ASSD (b) on DOX Ctrl, EO-DOX, and DEB's vessel skeleton extraction results. The central lines demonstrate the median values, the yellow points show the average values, the boxes represent the interquartile ranges, the whiskers exhibit the smallest and largest values, and the red points mark the outliers. Two-sided paired t-test was performed to compare groups (n=20 replicates). *P < 0.05, **P < 0.01, and ***P < 0.001.

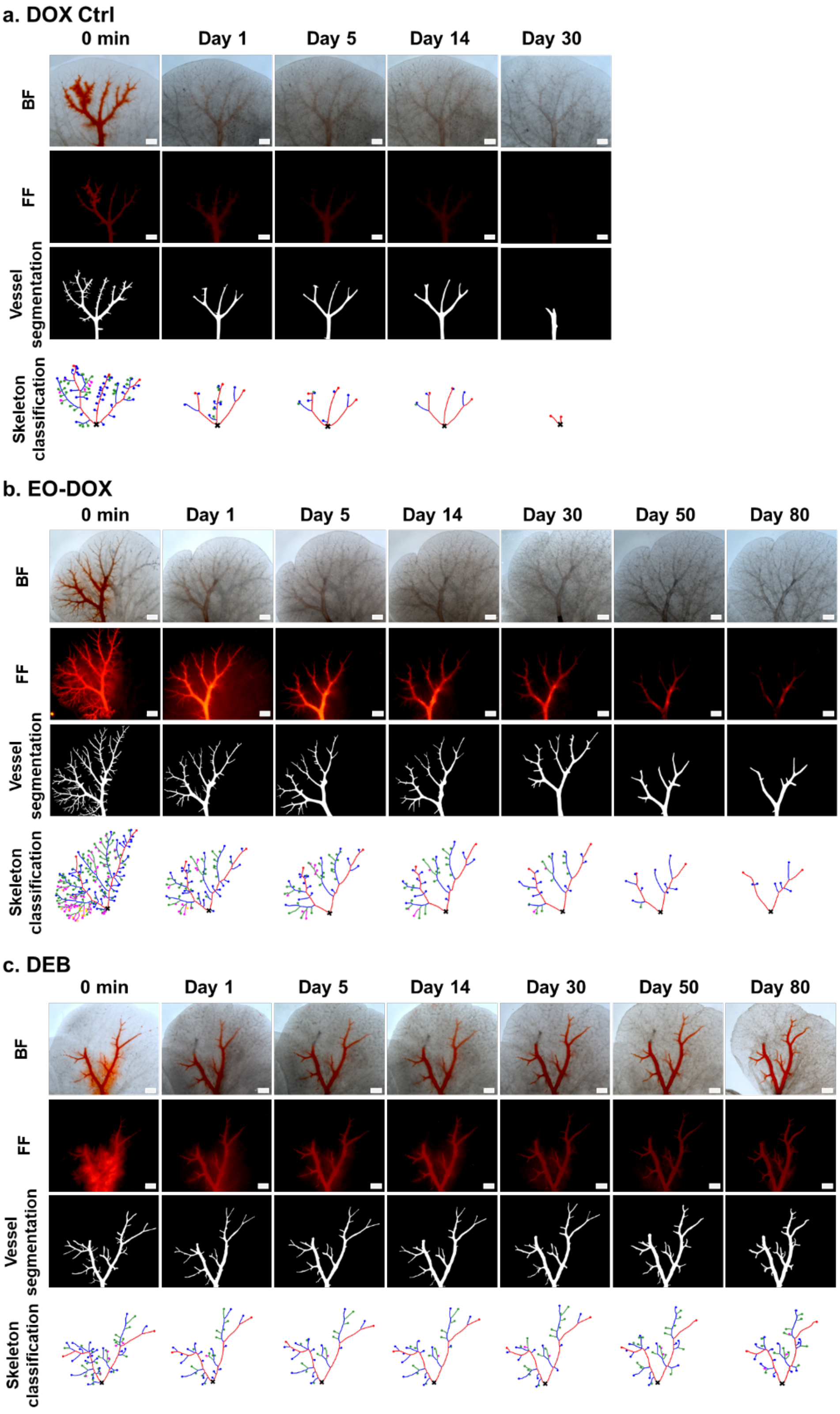

Fig. S4. Bright-field (BF) and fluorescent (FF) microscopy images of drug release behaviors with time for three drug formulations: i.e. DOX Ctrl (**a**), EO-DOX (**b**), and DEB (**c**). Vessel segmentation and skeleton classification of associated drug-containing vessels were also provided (bottom two rows in **a-c**). Scale bar: 2 mm (**a-c**).

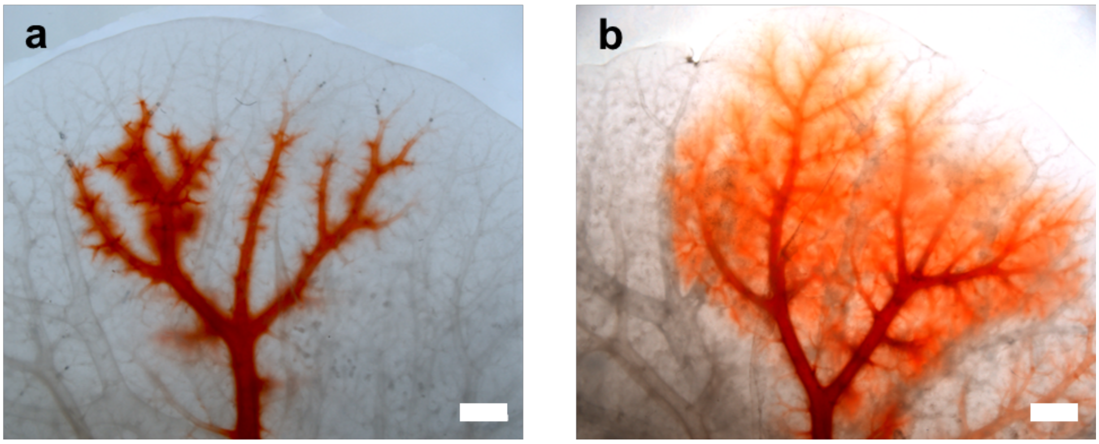

Fig. S5. Microscopy images of chemoembolization in DLM model right after injection with DOX Ctrl. The injection process was carefully performed at a slow speed to maintain the drug solution inside Levels P_1-P_3's vessel branches (**a**). Further injection into Levels P_4-P_5's vessel branches could easily cause severe extravascular perfusion (**b**). Scale bar: 2 mm (**a, b**).

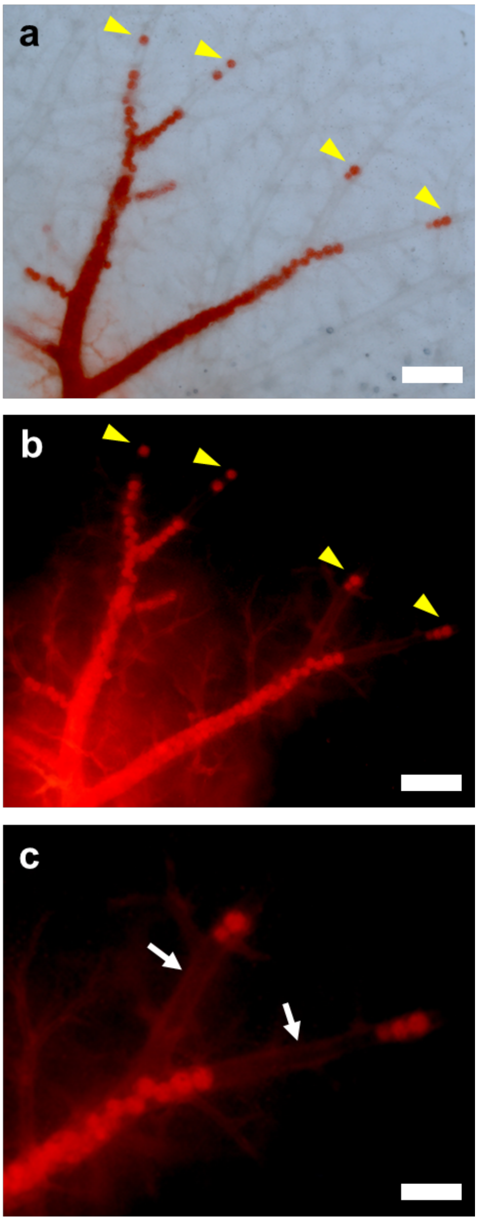

Fig. S6. Bright-field (a) and fluorescent (b, c) microscopy images of chemoembolization in DLM model right after injection with DEB. The drug-loaded microspheres were located inside Levels P1-P3's vessel branches with the embolization end-points dictated by the particle size and vessel dimension. The fluorescent image (**b, c**) was obtained using a Cy3 (530–560 nm excitation/573–647 nm emission) filter. Some disconnected embolic particles were marked by yellow triangles (**a, b**), and the diffused drug inside the gap between the disconnected embolic particles and the main embolized vessel was highlighted by white arrows (**c**). Scale bars: 1 mm (**a, b**), 500 µm (**c**).

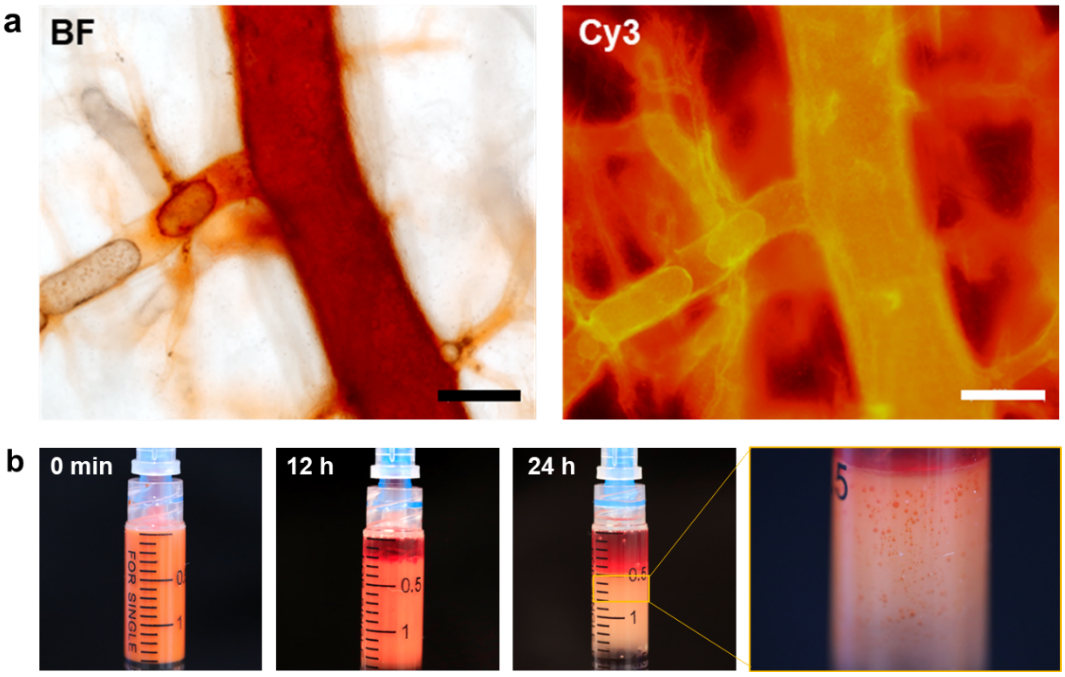

Fig. S7. Phase separation of EO-DOX observed in DLM model (a) and syringe (b). Scale bar: 500 μm (**a**).

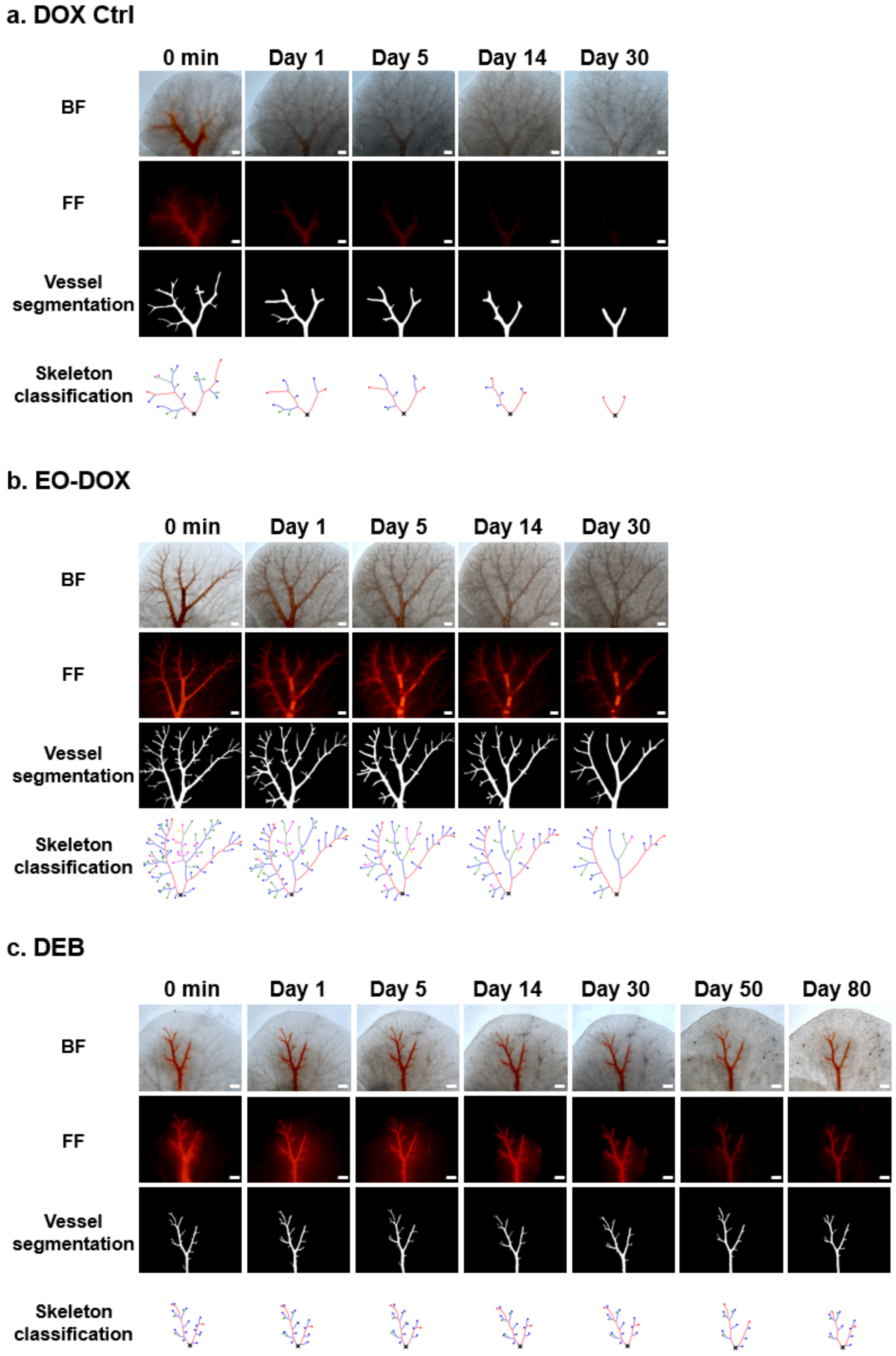

Fig. S8. Duplicate tests of TACE performances: i.e. DOX Ctrl (a), EO-DOX (b) and DEB (c). Bright-field (BF) and fluorescent (FF) microscopy images of each formulation were exhibited. Vessel segmentation and skeleton classification were also provided. Scale bar: 2 mm (**a-c**).

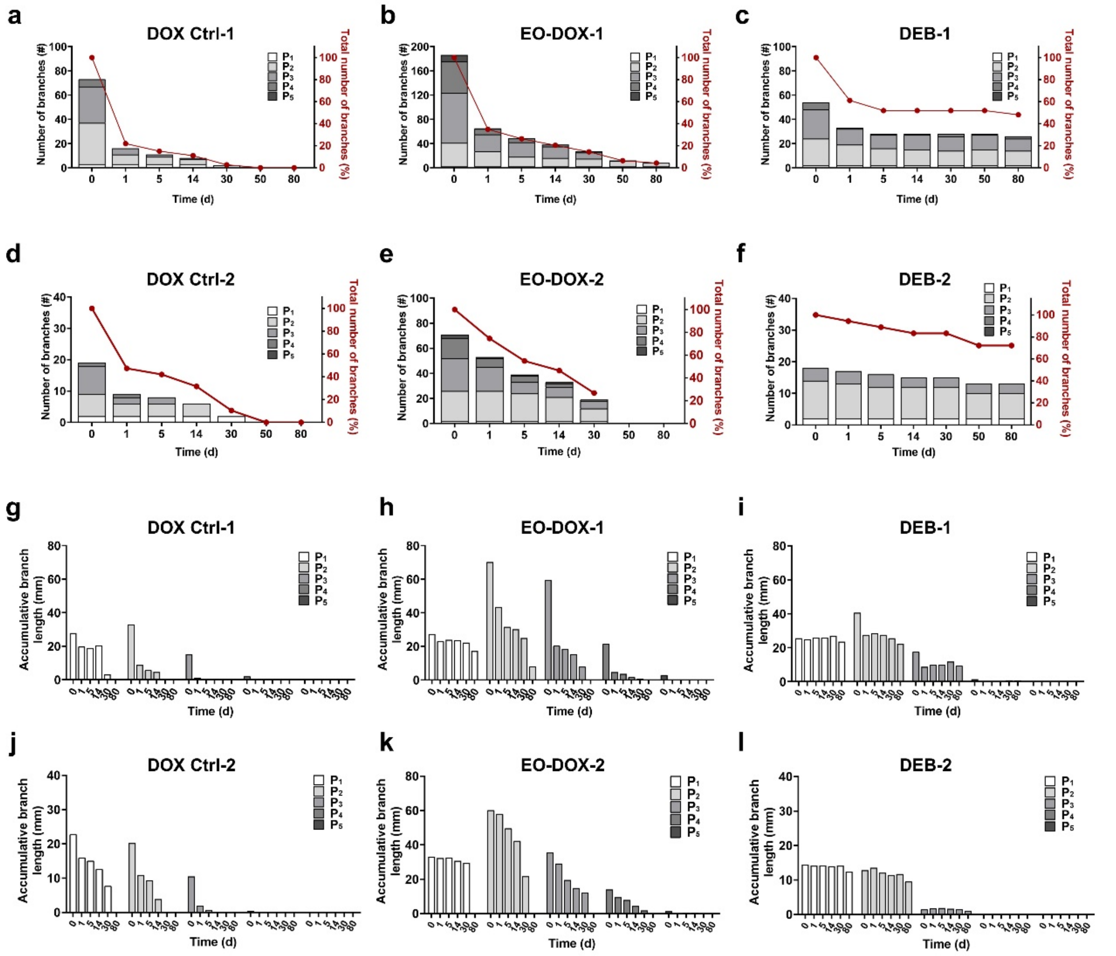

Fig. S9. Comparison of branch number (a-f) and accumulative branch length (g-l) of drug containing vessels in duplicate tests (test 1: a-c, g-i; test 2: d-f, j-l). Three drug formulations were characterized: i.e. DOX Ctrl (**a, d, g, j**), EO-DOX (**b, e, h, k**) and DEB (**c, f, i, l**).

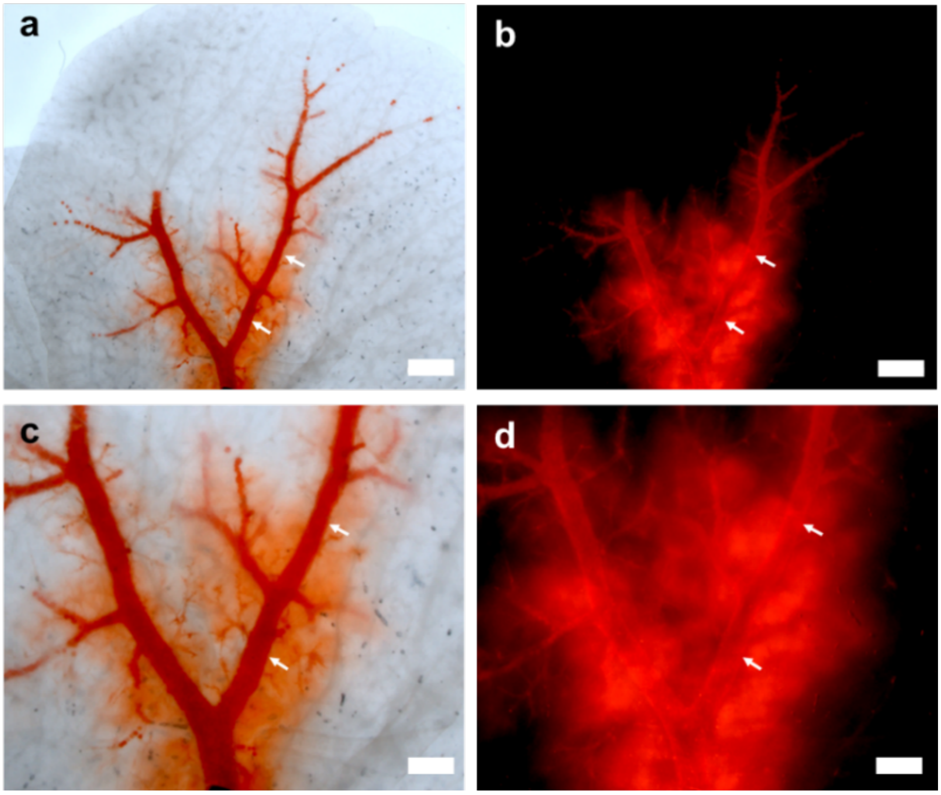

Fig. S10. Detection of the vessel wall of drug containing branches. (c, d) provide the enlarged views for (**a, b**), respectively. Two locations of drug-containing vessel wall were highlighted by white arrows. Scale bars: 2 mm (**a, b**), 1 mm (**c, d**).

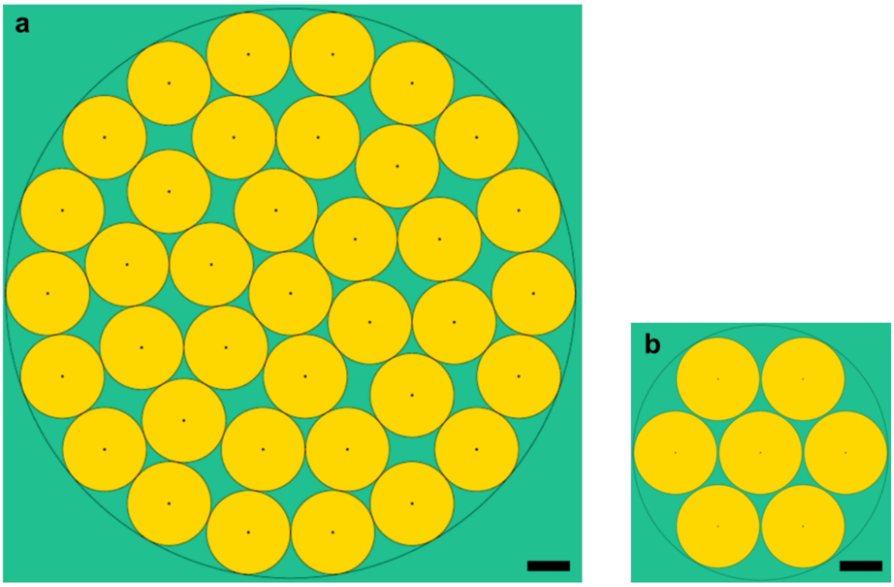

Fig. S11. Estimation of DEB microsphere quantity compacted inside vessels with different sizes: 666 µm (**a**) and 301 µm (**b**). The estimation was performed online: http://hydra.nat.unimagdeburg.de/packing/cci/cci.html. The diameter of the microsphere is 98 µm. Scale bar: 50 µm.

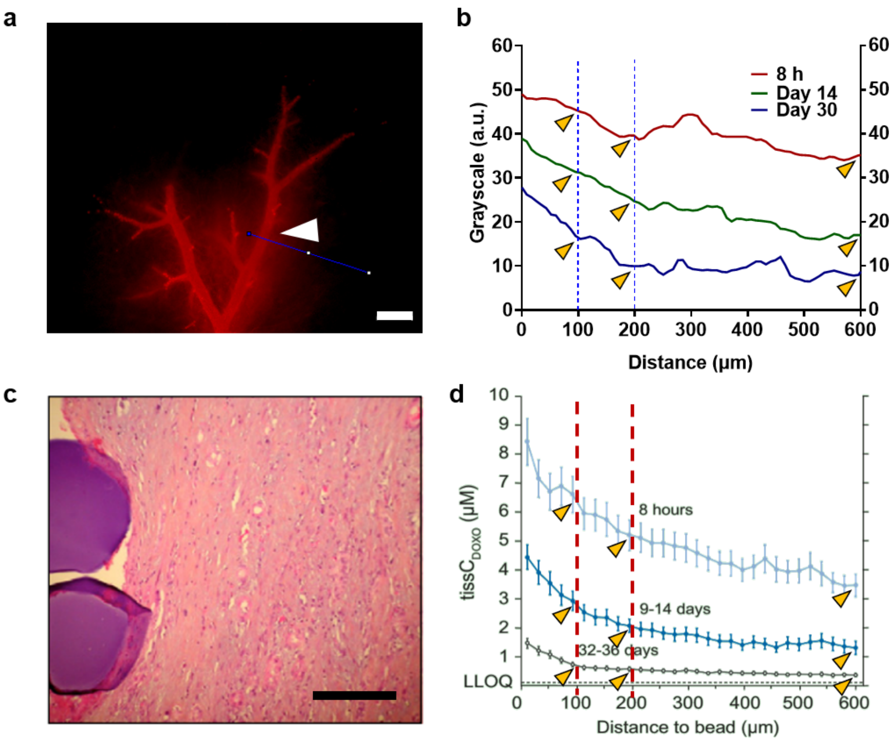

e

Location	G_n/G_0 (DLM) or C_n/C_0 (Namur et al.)					
	8 h		Day 14		Day 30	
	DLM	Namur et al.	DLM	Namur et al.	DLM	Namur et al.
100 μm	91.8%	74.5%	80.3%	61.1%	58.3%	44.7%
200 μm	79.9%	60.8%	63.1%	44.4%	35.9%	38.7%
600 μm	71.4%	41.2%	43.4%	29.6%	22.6%	22.0%

Fig. S12. IVIVC establishment based on DLM model (a, b) and in-human results (c, d). According to the fluorescent image of DEB embolized DLM model (**a**), extravascular drug diffusion was analyzed by recording the grayscale vs. extravascular drug diffusion depth along with the blue line (**b**). The histological sections of HCC (**c**) were analyzed by Namur et al. to derive the correlation of extravascular drug concentration vs. extravascular drug diffusion (**d**) based on the assumption of the assumption of linear relationship of doxorubicin fluorescent signal with the drug concentration. The extravascular diffusion depth of three locations (100 μm, 200 μm and 600 μm) was assessed to derive the ratio of the grayscale (G_n/G_0) or the ratio of drug concentration (C_n/C_0) for IVIVC establishment (**e**). Yellow triangles highlight the different locations selected for analysis (**b, d**). Scale bar: 2 mm (**a**), and 100 μm (**c**). (**c, d**) are reproduced from Namur et al. by permission of Elsevier.[1]

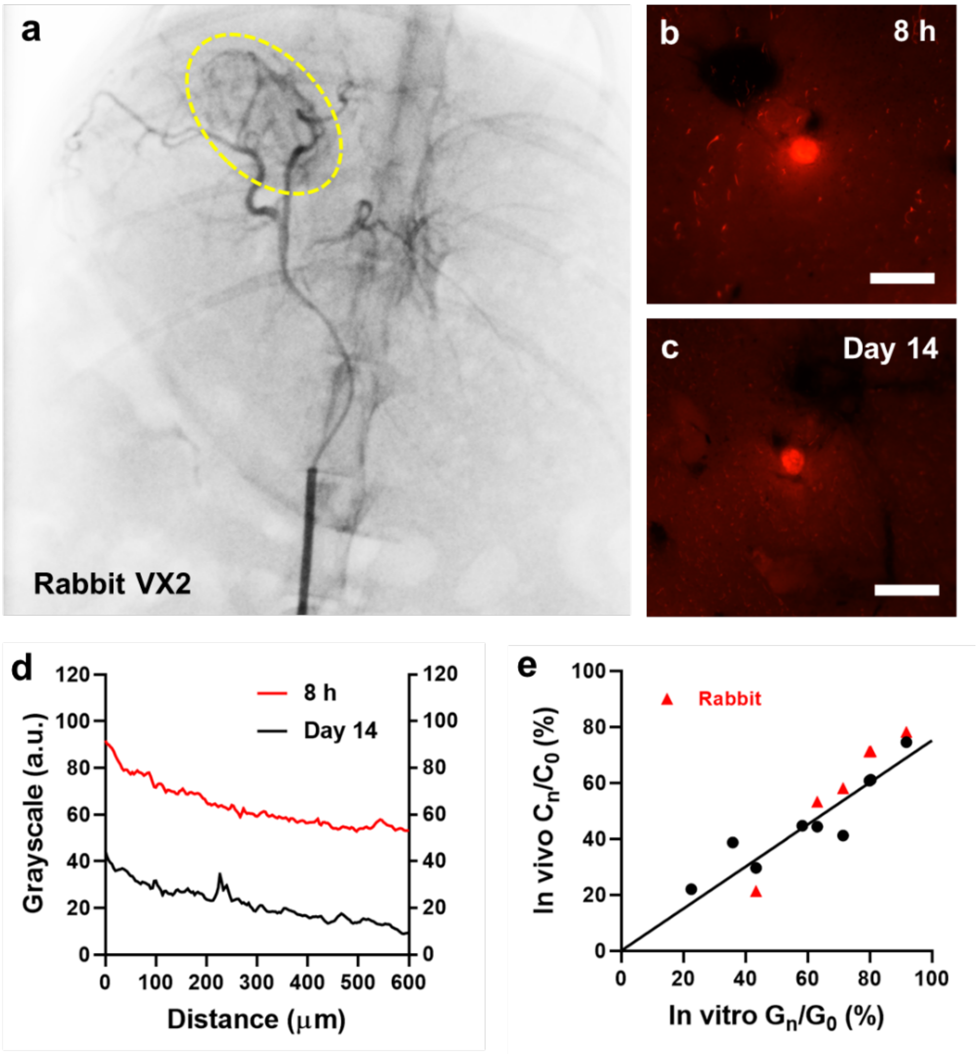

Fig. S13. DEB-TACE performance in a rabbit VX2 liver cancer model (digital subtraction angiography image, a). Doxorubicin-loaded DEB was clearly observed in the tumor sections embolized for 8 h (**b**) and 14 d (**c**), respectively. Extravascular drug diffusion (**d**) was analyzed and well fitted in the IVIVC curve obtained in Fig. 4I (**e**). Yellow dashed ellipse marked the tumor planted in the rabbit liver lobe. Scale bar: 500 μm.

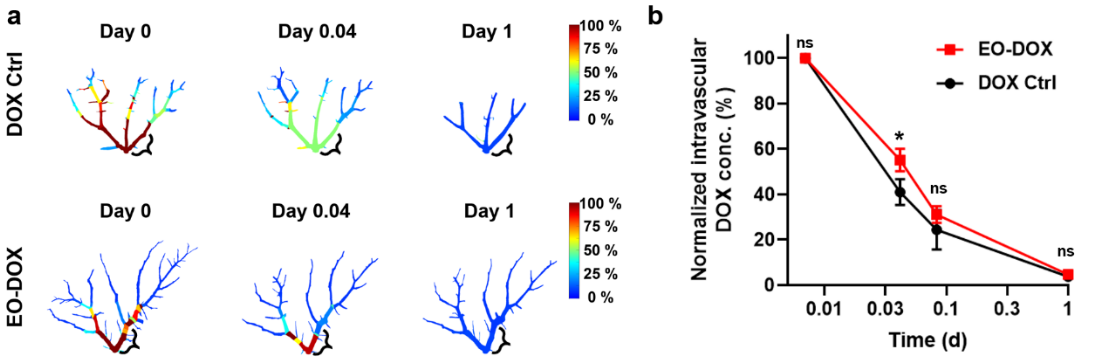

Fig. S14. In the main vessels of Level 1 close to the root, EO-DOX exhibited similar drug release behavior to DOX Ctrl for the first 24 h, showing slightly higher drug concentration only on Day 0.04 (a). Curly brackets marked the location of the main vessels of Level 1 close to the root (**a**) for the analyses of normalized intravascular drug concentration with time (**b**). Data are shown as means ± SEM (n=10 replicates) (**b**). *P < 0.05.

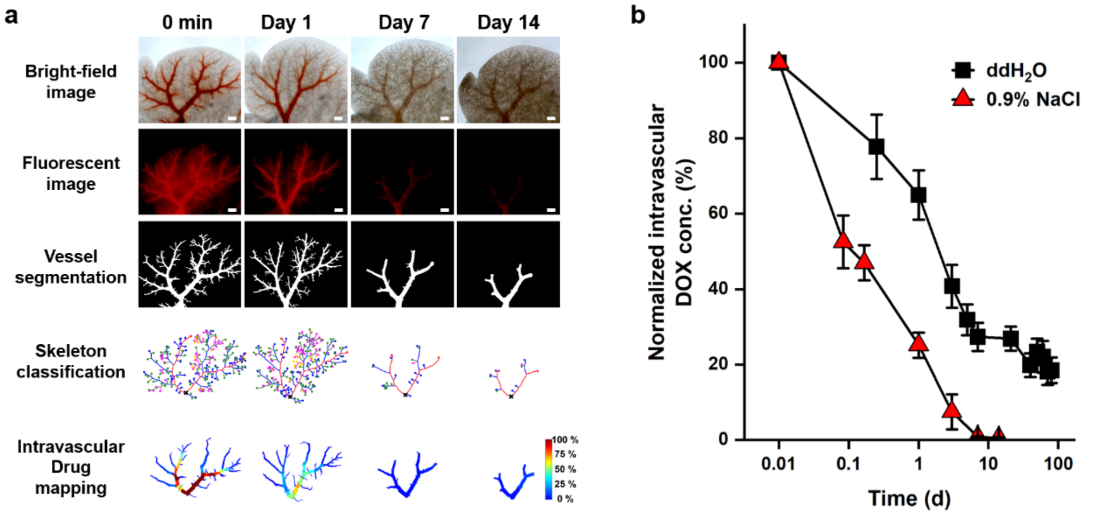

Fig. S15. Drug release from DEB using 0.9% NaCl perfusion in DLM model. a. The drug release behavior recorded in bright-field and fluorescent images was analyzed in terms of vessel segmentation, skeleton classification, and intravascular drug mapping. **b**. Comparison of intravascular doxorubicin retention using different perfusion buffers. Scale bar: 2 mm (**a**).

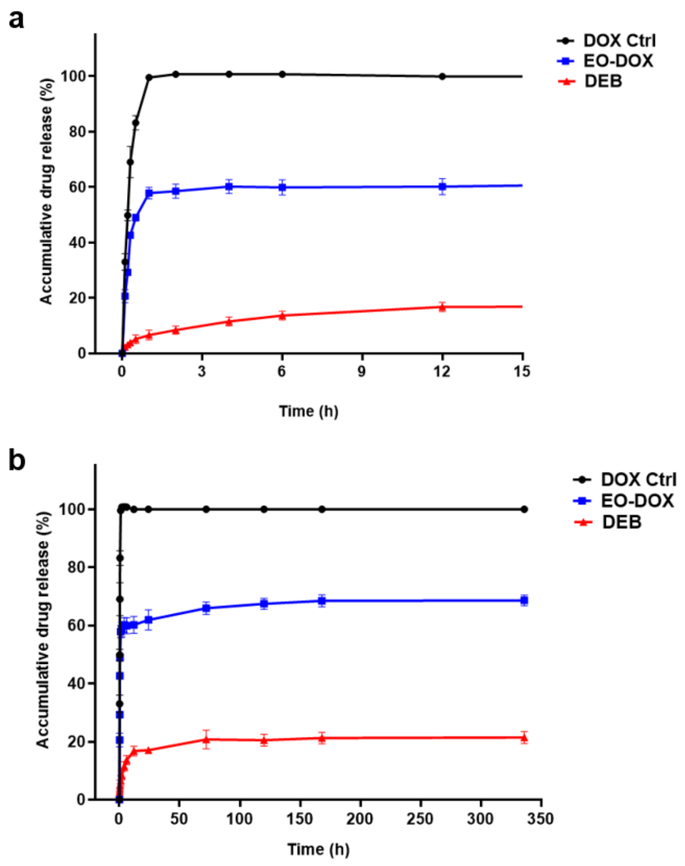

Fig. S16. Drug release profiles of DOX Ctrl, EO-DOX and DEB performed in dialysis bags using PBS buffer (pH = 7.4). Both EO-DOX and DEB exhibited an initial drug burst within 12 h (**a**) but the remaining drug could be barely released from the dialysis bags at the latter time points (**b**).

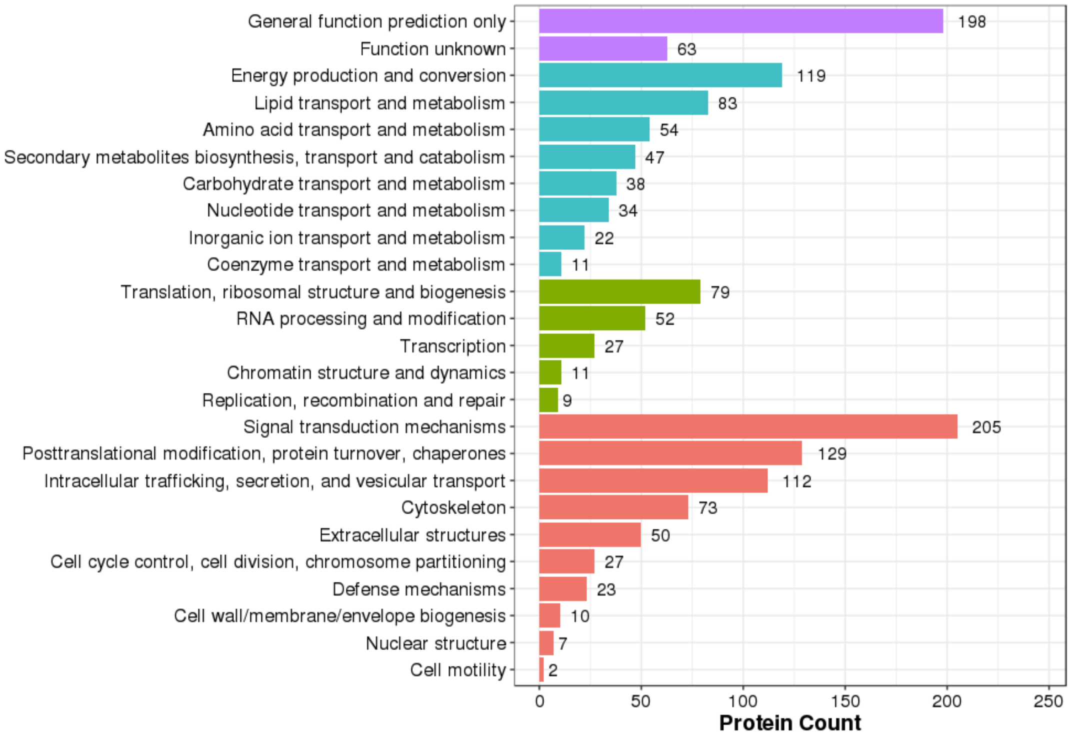

Fig. S17. KOG analysis of differential expression proteins between S2 group and fresh liver.

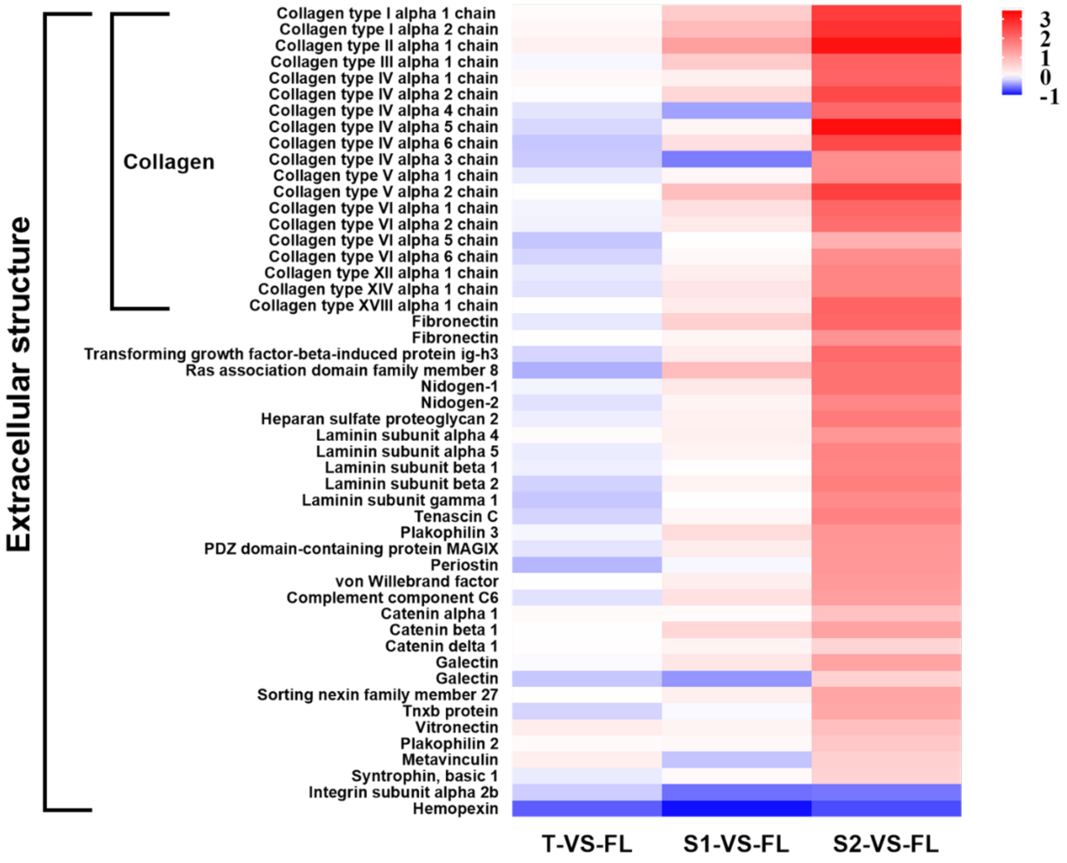

Fig. S18. Cluster analysis of 50 identified differential expressed proteins between S2 group and fresh liver that were classified as extracellular matrix according to KOG. The color bar represents the log2 fold change of protein level. Blue indicates down-regulation, red indicates up-regulation, white indicates no detectable expression change.

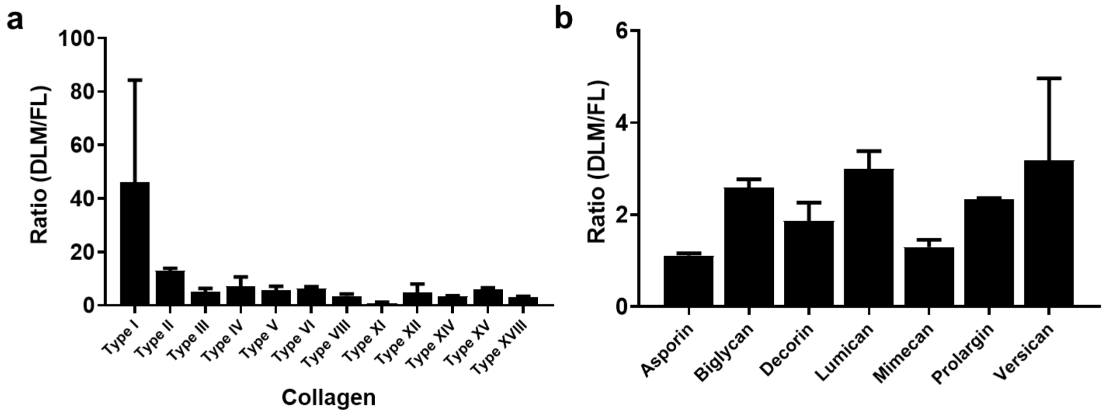

Fig. S19. Ratios of decellularized to fresh liver tissues of collagens (a) and proteoglycans (b) identified by proteomic analyses. Data are shown as means ± SD (n=3 replicates).

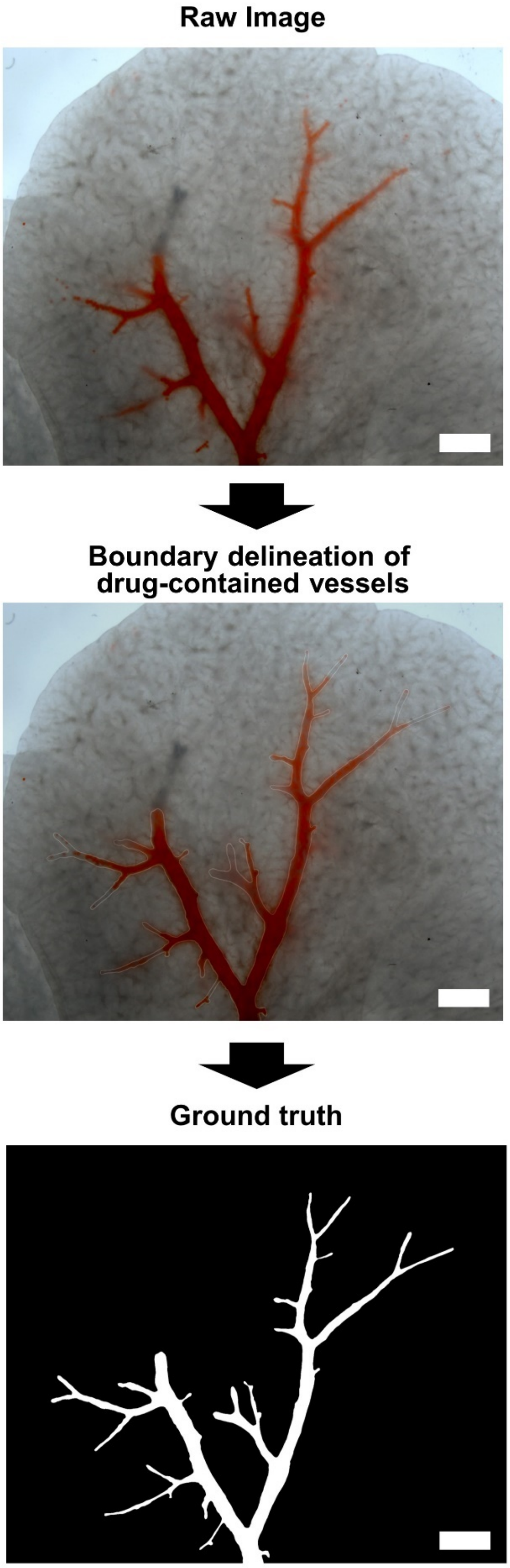

Fig. S20. Demonstration of ground truth image obtained through boundary delineation of drug-contained vessels through a Procreate® App with an Apple Pencil in iPad. Scale bar: 2 mm.

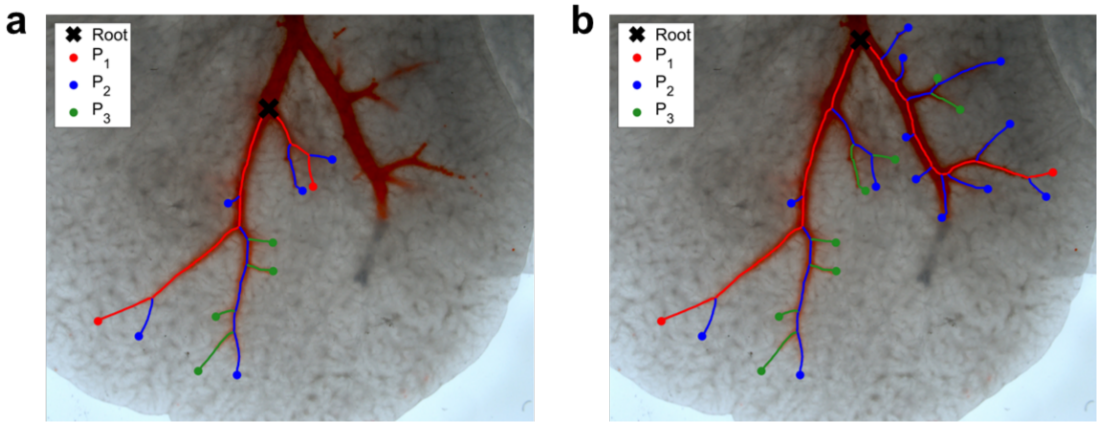

Fig. S21. Demonstration of manual correction of a root node of vessel skeleton. The root node of (**a**) was corrected to make sure to incorporate all targeted drug-containing vessel branches for analyses (**b**).

Table S1. Branch number of DOX Ctrl analyzed in Fig. 3b.

Time (d) \ Branch number (#)	P_1	P_2	P_3	P_4	P_5
**0**	3	34	30	6	0
**1**	3	8	5	0	0
**5**	3	6	2	0	0
**14**	3	4	1	0	0
**30**	2	0	0	0	0
**80**	0	0	0	0	0

Table S2. Branch number of EO-DOX analyzed in Fig. 3c.

Time (d) \ Branch number (#)	P_1	P_2	P_3	P_4	P_5
**0**	2	39	83	51	11
**1**	2	25	28	9	1
**5**	2	16	24	7	0
**14**	2	14	19	3	0
**30**	2	13	10	2	0
**80**	2	9	0	0	0

Table S3. Branch number of DEB analyzed in Fig. 3d.

Time (d) \ Branch number (#)	P_1	P_2	P_3	P_4	P_5
**0**	2	22	24	6	0
**1**	2	17	13	1	0
**5**	2	14	11	1	0
**14**	2	13	12	1	0
**30**	2	12	12	2	0
**80**	2	13	12	1	0

Table S4. Total branch length of DOX Ctrl analyzed in Fig. 3e.

Branch Length (mm) / Time (d)	P_1	P_2	P_3	P_4	P_5
**0**	27.802	32.974	15.069	1.957	0.000
**1**	19.716	8.776	1.181	0.000	0.000
**5**	18.810	5.802	0.336	0.000	0.000
**14**	20.250	4.595	0.233	0.000	0.000
**30**	3.009	0.000	0.000	0.000	0.000
**80**	0.000	0.000	0.000	0.000	0.000

Table S5. Total branch length of EO-DOX analyzed in Fig. 3f.

Branch Length (mm) / Time (d)	P_1	P_2	P_3	P_4	P_5
**0**	27.112	70.086	59.560	21.517	2.940
**1**	23.060	43.267	20.302	4.629	0.293
**5**	23.853	31.517	18.345	3.578	0.000
**14**	23.629	30.095	15.190	1.767	0.000
**30**	22.164	24.897	7.862	0.750	0.000
**80**	17.147	7.905	0.000	0.000	0.000

Table S6. Total branch length of DEB analyzed in Fig. 3g.

Branch Length (mm) / Time (d)	P_1	P_2	P_3	P_4	P_5
**0**	25.560	40.543	17.603	1.379	0.000
**1**	24.931	27.276	8.690	0.466	0.000
**5**	25.888	28.414	9.845	0.155	0.000
**14**	25.931	27.448	10.026	0.172	0.000
**30**	26.776	25.526	11.771	0.784	0.000
**80**	23.552	22.122	9.310	0.575	0.000

Table S7. Information of 50 identified differentially expressed proteins associated with extracellular structures based on eukaryotic orthologous group (KOG) database in S2-VS-FL group.

Protein ID	Protein Descriptive
**CO1A1_RAT**	Collagen type I alpha 1 chain
**A0A0G2KAN1_RAT**	Collagen type I alpha 2 chain
**F1LRM7_RAT**	Collagen type II alpha 1 chain
**CO3A1_RAT**	Collagen type III alpha 1 chain
**F1MA59_RAT**	Collagen type IV alpha 1 chain
**F1M6Q3_RAT**	Collagen type IV alpha 2 chain
**A0A096MIZ6_RAT**	Collagen type IV alpha 4 chain
**F1LUN5_RAT**	Collagen type IV alpha 5 chain
**A0A0G2K601_RAT**	Collagen type IV alpha 6 chain
**Q63122_RAT**	Collagen type IV alpha 3 chain
**CO5A1_RAT**	Collagen type V alpha 1 chain
**F1LQ00_RAT**	Collagen type V alpha 2 chain
**D3ZUL3_RAT**	Collagen type VI alpha 1 chain
**F1LNH3_RAT**	Collagen type VI alpha 2 chain
**F1LZF4_RAT**	Collagen type VI alpha 5 chain
**D3ZL10_RAT**	Collagen type VI alpha 6 chain
**A0A0G2KAJ7_RAT**	Collagen type XII alpha 1 chain
**D3ZZT9_RAT**	Collagen type XIV alpha 1 chain
**F1LR02_RAT**	Collagen type XVIII alpha 1 chain
**FINC_RAT**	Fibronectin
**F1LST1_RAT**	Fibronectin
**D4A8G5_RAT**	Transforming growth factor-beta-induced protein ig-h3
**D4AAU5_RAT**	Ras association domain family member 8
**F1LM84_RAT**	Nidogen-1
**A0A0G2K3C8_RAT**	Nidogen-2
**F1LTJ5_RAT**	Heparan sulfate proteoglycan 2
**F1LTF8_RAT**	Laminin subunit alpha 4
**F1MAN8_RAT**	Laminin subunit alpha 5
**D3ZQN7_RAT**	Laminin subunit beta 1
**LAMB2_RAT**	Laminin subunit beta-2
**F1MAA7_RAT**	Laminin subunit gamma 1
**B2LYI9_RAT**	Tenascin C
**D3ZJ50_RAT**	Plakophilin 3
**MAGIX_RAT**	PDZ domain-containing protein MAGIX
**A0A097BW25_RAT**	Periostin
**F1M957_RAT**	von Willebrand factor
**F1M7F7_RAT**	Complement component C6
**Q5U302_RAT**	Catenin (Cadherin associated protein), alpha 1
**CTNB1_RAT**	Catenin beta-1

**D3ZZZ9_RAT**	Catenin (Cadherin associated protein)
**Q6P7Q6_RAT**	Galectin
**V5QSV9_RAT**	Galectin
**G3V8U4_RAT**	Sorting nexin family member 27
**B4F793_RAT**	Tnxb protein
**Q3KR94_RAT**	Vitronectin
**F1M7L9_RAT**	Plakophilin 2
**R9PXU6_RAT**	Metavinculin
**D3ZWC6_RAT**	Syntrophin, basic 1
**D3ZAC0_RAT**	Integrin subunit alpha 2b
**HEMO_RAT**	Hemopexin

Table S8. Information of 14 collagens and 8 proteoglycans identified in decellularized liver samples.

**Collagen**		**Proteoglycan**	
**Protein ID**	**Descriptive**	**Protein ID**	**Descriptive**
**A0A0G2KAN1_RAT**	Type I	**D3ZFC3_RAT**	Asporin
**F1LRM7_RAT**	Type II	**D3ZVB7_RAT**	Biglycan
**CO3A1_RAT**	Type III	**PGS1_RAT**	Decorin
**F1LUN5_RAT**	Type IV	**PGS2_RAT**	Lumican
**F1LQ00_RAT**	Type V	**Q5XIH1_RAT**	Mimecan
**D3ZUL3_RAT**	Type VI	**PRELP_RAT**	Osteomodulin
**D4AC70_RAT**	Type VIII	**ATS4_RAT**	Prolargin
**A0A0G2K7A5_RAT**	Type X	**OMD_RAT**	Versican core protein
**COBA1_RAT**	Type XI		
**A0A0G2KAJ7_RAT**	Type XII		
**D3ZZT9_RAT**	Type XIV		
**F1LPD0_RAT**	Type XV		
**F1LR02_RAT**	Type XVIII		
**D3ZCQ0_RAT**	Type XIX		

Reference:

1. Namur, J. *et al.* Embolization of hepatocellular carcinoma with drug-eluting beads: doxorubicin tissue concentration and distribution in patient liver explants. *J Hepatol* **55**, 1332-1338 (2011).
